# Complete Genome Sequence and Comparative Genomics of *Acetobacter cerevisiae* KSO5 (KACC 92352P) Provide Genome-Based Insights into Acid Tolerance

**DOI:** 10.3390/microorganisms14051128

**Published:** 2026-05-15

**Authors:** Sun Hee Kim, Dae Gyu Choi, Dong Min Han, SeongEui Yoo, Jin Ju Park, Chan-Woo Kim, So-Young Kim

**Affiliations:** 1 Fermented and Processed Food Research Division, Department of Food Sciences, National Instituted of Crop and Food Science, Rural Development Administration, Wanju 55365, Republic of Korea; sunheekim00@korea.kr (S.H.K.); dbtjddml@korea.kr (S.Y.); waemma25@korea.kr (J.J.P.); kcw5142@korea.kr (C.-W.K.); 2 Department of Life Science, Chung-Ang University, Seoul 06974, Republic of Korea; chleorb21@cau.ac.kr (D.G.C.); gonigori@naver.com (D.M.H.)

**Keywords:** *Acetobacter cerevisiae*, complete genome, oxidative fermentation, ethanol stress, vinegar fermentation

## Abstract

*Acetobacter cerevisiae* KSO5 is an indigenous strain isolated from Korean fruit vinegar and is a potential starter candidate for vinegar fermentation. Here, we report the first complete circular genome of KSO5, comprising a 3.3 Mb chromosome and two plasmids encoding 2898 genes. Core-genome phylogeny clearly placed KSO5 within the *A. cerevisiae* clade, supported by ANI (97%) and dDDH (71%) values. Comparative analysis with seven draft *A. cerevisiae* genomes identified strain-specific genomic islands, mobile genetic elements, and plasmid-borne modules potentially related to genetic stability. Comparative COG profiling suggested enhanced potential for carbohydrate utilization, redox balancing, membrane transport, and stress adaptation within a conserved *Acetobacter* genomic background. The genome encoded a periplasmic oxidative fermentation system, including membrane-bound pyrroloquinoline quinone-dependent alcohol dehydrogenase and molybdopterin-dependent aldehyde dehydrogenase, together with predicted acetate-handling routes that may reduce intracellular acetate accumulation. Consistent with these features, KSO5 maintained growth and titratable acidity production up to 9% ethanol, with the strongest performance at 7–9% ethanol, whereas both traits declined markedly at 10% ethanol. In 5% ethanol medium, KSO5 also showed high ethanol consumption, comparable to that of *A. pasteurianus* LMG 1262 and higher than that of most reference strains. These findings link the genomic features of KSO5 to efficient ethanol oxidation, sustained acidification, and stable growth, supporting its potential as a starter strain for vinegar fermentation.

## 1. Introduction

Vinegar production depends on the remarkable physiology of acetic acid bacteria (AAB), which oxidize reduced substrates in the periplasm and channel electrons to the respiratory chain while tolerating combined stresses of ethanol, oxygen gradients, and high acetic-acid levels [1]. Central to this lifestyle are membrane-bound dehydrogenase systems—most prominently a pyrroloquinoline quinone (PQQ)-dependent alcohol dehydrogenase (PQQ-ADH) and, typically, a membrane-bound molybdopterin (Mo)-dependent acetaldehyde dehydrogenase (Mo-ALDH; AldFGH-type)—that catalyze the two-step conversion of ethanol to acetate at the periplasmic face of the inner membrane, routing reducing equivalents into the ubiquinone pool and largely decoupling carbon oxidation from cytosolic NAD(H) balance, thereby enabling rapid, surface-confined oxidative fermentation [1,2,3]. The resulting electron flux is dissipated via branched terminal oxidases (e.g., bo_3_- and bd-type ubiquinol oxidases) and auxiliary respiratory modules, together sustaining ATP generation and redox homeostasis under acidic and microaerobic conditions typical of vinegar fermentations [4].

There has been continued interest in identifying acetic acid bacterial strains with desirable technological and functional properties for food fermentation, as these bacteria play key roles in vinegar production and other oxidative fermentation processes. At the same time, the need to secure indigenous microbial resources has increased, further highlighting the importance of domestically sourced AAB with potential industrial applicability. In this context, we isolated and screened AAB from farmhouse vinegars using a CaCO_3_-containing medium to identify functionally relevant strains. Among the isolates, strain KSO5 was selected because it showed a clear halo-forming, acid-producing phenotype on CaCO_3_-containing medium under ethanol-supplemented aerobic conditions and had previously been characterized as a functional AAB with antibacterial, antioxidant, antihypertensive, and antidiabetic activities [5]. These characteristics suggest that KSO5 may represent a valuable indigenous *Acetobacter* strain with considerable fermentation-related potential.

While *Acetobacter cerevisiae* has been recurrently isolated from food fermentations, a closed, reference-quality genome sequence has not been reported for this species in the literature, and publicly available resources have largely consisted of draft assemblies. Accordingly, comparative inferences have often drawn on related AAB such as *Gluconobacter oxydans*, which lacks respiratory complex I and exhibits a distinct respiratory architecture [6]. However, such cross-species inference cannot fully resolve the genome organization, mobile genetic elements, plasmid-associated modules, or stress-response loci that may directly contribute to the physiology and fermentation-relevant traits of *A. cerevisiae* itself.

In AAB, tolerance to ethanol, acetic acid, and other fermentation-associated stresses is a critical determinant of strain robustness, and these phenotypes are often shaped by genome context, including strain-specific genes, structural variation, mobile elements, and plasmid-borne functions. A complete genome is therefore required to accurately define these features and to establish a robust framework linking genotype to phenotype within the species.

In this study, we generated a complete closed circular genome of *A. cerevisiae* strain KSO5 and used it as a species-level reference framework for comparative analysis with seven publicly available draft genomes of *A. cerevisiae*. We hypothesized that the acid-producing and fermentation-relevant phenotype of KSO5 is supported by conserved AAB oxidative-fermentation machinery together with strain-specific chromosomal and plasmid-associated features involved in ethanol and acid stress adaptation. To test this hypothesis, we combined high-confidence structural and functional annotation with comparative genomics to identify genomic islands, other mobile genetic elements, conserved stress-response loci, single-nucleotide polymorphisms, and plasmid-related protein modules. To further connect genotype with phenotype, we evaluated KSO5 under ethanol exposure conditions and compared its performance with taxonomically and technologically informative reference strains. Specifically, phenotypic benchmarking was performed using the *A. cerevisiae* type strain LMG 1625 as the primary conspecific reference and representative vinegar-associated *Acetobacter* strains, including *A. pasteurianus* type strain LMG 1262, *A. aceti* type strain NBRC 14818, and *A. malorum* type strain LMG 1746. Together, these analyses provide a genome-enabled, systems-level view of *A. cerevisiae* physiology, linking respiratory architecture, carbohydrate oxidation, and stress tolerance to genotype variation, and establishing a foundation for hypothesis-driven functional studies of fermentation-relevant traits.

## 2. Materials and Methods

### 2.1. Bacterial Isolation

Samples were collected from farm-produced omija (magnolia berry, *Schisandra chinensis*) fruit vinegar in Gyeonggi Province, South Korea. A 100–200 µL aliquot of the vinegar sample was spread onto YGC agar plates and incubated at 30 °C for 3 days under aerobic conditions. YGC agar medium was prepared with 0.5% (*w*/*v*) yeast extract, 3.0% (*w*/*v*) glucose, 1.0% (*w*/*v*) CaCO_3_, and 2.0% (*w*/*v*) agar, sterilized, cooled to approximately 50 °C, and then supplemented with 4.0% (*v*/*v*) ethanol before pouring into plates. Representative colonies, characterized by a clear halo indicating the dissolution of CaCO_3_ in the medium due to acetic acid production, were selected from the plates. These colonies were further purified using fresh YGC agar plates and stored at −80 °C in YG broth containing 80% glycerol for subsequent analysis.

### 2.2. Phenotyping Under Ethanol Stress

Acetic acid bacterial strains were cultured aerobically in yeast extract–glucose medium supplemented with 1% (*v*/*v*) acetic acid and ethanol at final concentrations of 5–10% (*v*/*v*). Cultures were prepared in 250 mL Erlenmeyer flasks containing 50 mL of medium, standardized to an inoculum density of OD_660_ = 0.5, and inoculated at a ratio of 1:1000 (*v*/*v*). Incubation was performed at 30 °C and 150 rpm for up to 10 days. Growth (OD_660_), titratable acidity, and ethanol consumption capacity were measured at the designated sampling time points. Unless otherwise stated, quantitative phenotypic assays were conducted using four independent biological replicates derived from two independent experiments, with two biological cultures per experiment (*n* = 4). Statistical differences among strains were analyzed by one-way ANOVA followed by Dunnett’s multiple-comparison test using *A. cerevisiae* KSO5 as the reference strain. Detailed procedures, calculation formulas, and instrument information are provided in the Supplementary Methods.

### 2.3. Characterization of Bacterial Isolates

The morphological characteristics of the bacterial isolates were observed using a Leica optical DM500 or stereo microscope EZ4 (Leica Microsystems, Wetzlar, Germany) and a ZEISS Gemini Scanning electron microscope (SEM) 300 (ZEISS, Jena, Germany) after culturing the AAB on YGC solid medium at 30 °C for three days. The Gram reaction was determined using the standard Gram staining method. To further assess the physiological, biochemical, and enzymatic activities of the isolates, experiments were conducted using the API ZYM test kit (25200, BioMérieux, Marcy-l’Étoile, France) according to the manufacturer’s instructions.

### 2.4. Phylogenetic Analysis

The 16S rRNA gene was amplified using primers 27F (5′-AGAGTTTGATCCTGGCTCAG-3′) and 1492R (5′-GGTTACCTTGTTACGACTT-3′) and the amplicons were sequenced. Initial taxonomic assignment was performed using BLASTn (Mega 6.06) against the NCBI GenBank database (NCBI BLAST+ v2.17.0.).

For additional taxonomic comparison, 16S rRNA gene-based phylogenetic analyses were performed using representative sequences obtained from public sequence records or retrieved from genome assemblies by BLAST (Mega 7) searches when registered 16S rRNA gene sequences were unavailable. *Lichenicoccus roseus* KCTC 72321 was included as an outgroup. The 16S rRNA gene sequences were aligned using Infernal v1.14, and phylogenetic trees were reconstructed in MEGA 7 using neighbor-joining, maximum-likelihood, and maximum-parsimony methods. The neighbor-joining tree was constructed using the Maximum Composite Likelihood method, the maximum-likelihood tree was inferred using the Tamura–Nei model [7], and the maximum-parsimony tree was generated using the subtree-pruning-regrafting search method. Branch support was assessed with 1000 bootstrap replicates.

### 2.5. Genome Sequencing and Assembly

Genomic DNA from the KSO5 strain was extracted using the QIAamp DNA Mini Kit (Qiagen, Valencia, CA, USA) following the manufacturer’s instructions. Whole-genome sequencing and analyses were performed by Macrogen (Seoul, Republic of Korea) in accordance with minimal standards for prokaryotic taxonomy. PacBio sequel subreads were assembled with the microbial assembly pipeline in SMRT Link v13.0.0.207600 (HGAP) using default parameters. For polishing, illumina paired-end raw reads were quality-filtered under Macrogen’s internal QC criterion designed to maximize the precision of read-based error correction: only reads with ≥90% of bases having a Phred quality score ≥30 (Q30; ~99.9% base-call accuracy) were retained, and reads failing this threshold were excluded to avoid spurious corrections during Pilon polishing. The assembly was then polished with Pilon v1.21 using the retained high-quality reads [8]. Gene prediction and primary annotation were performed with Prokka v1.14.6 (--compliant, --rnammer, --addgenes) [9], and protein-coding genes were functionally classified using the Clusters of Orthologous Groups (COGs) database. Assembly quality was documented by contiguity and completeness indicators: the final assembly deposited at NCBI under ASM4409463v1 (=GCA_044094635.1) comprises one circular chromosome (CP172014.1; 3,257,599 bp) and two small circular plasmids (CP172015.1, 4905 bp; CP172016.1, 4820 bp), yielding a contig N50 of 3,257,599 bp and an L50 of 1; NCBI lists the assembly level as “Complete genome.”

### 2.6. Comparative Genomics and Functional Analysis

Comparative genomic analyses were conducted at two hierarchical levels. For the acetic acid bacteria (AAB)-wide analysis, 39 genomes, including KSO5, were used as the ingroup dataset (Table 1). This dataset comprised complete genomes from the genera *Acetobacter*, *Gluconobacter*, *Gluconacetobacter*, and *Komagataeibacter* available in the NCBI Assembly database as of 11 February 2025. For species-level comparison, eight *Acetobacter cerevisiae* genomes were analyzed, including the complete genome of KSO5 and seven publicly available draft assemblies (Table 2). Genome annotation was performed using Prokka v1.14.6 [10], and the resulting GFF and predicted protein files were used for downstream comparative analyses.

For species-level pan-genome analysis of the eight *A. cerevisiae* genomes, Roary v3.13.0 [10] was used with a BLASTP (Roary v3.13.0) identity threshold of 90% using the options -i 90 -e -n. Core-gene alignments generated by Roary were used for phylogenomic reconstruction with FastTree v2.1.11 [11], and the resulting trees were visualized using iTOL v5 [12].

Core genome-based phylogenetic analysis was further performed using the 39 AAB genomes. *Lichenicoccus roseus* KCTC 72321 was included as an outgroup for tree rooting. GFF annotation files were used as input for Roary to identify orthologous gene clusters and generate a core-gene nucleotide alignment. Roary was executed using the following command:

roary -f ./ -i 50 -e -n -p 72 ./gff/*.gff

In this analysis, a minimum BLASTP version 2.15.0 identity threshold of 50% was applied, and core genes were defined according to the Roary default criterion as genes present in at least 99% of the analyzed genomes. This threshold was selected to retain broadly conserved orthologous genes across the phylogenetically diverse AAB genomes included in this study. The -e and -n options were used to generate a nucleotide alignment of core genes.

A maximum-likelihood phylogenetic tree was subsequently inferred from the core-gene alignment using IQ-TREE with the following command:

iqtree -s core_gene_alignment.fasta -st DNA -m MFP -bb 1000 -nt 72

The optimal nucleotide substitution model was automatically selected using ModelFinder Plus, and branch support was evaluated using 1000 ultrafast bootstrap replicates. The final phylogenetic tree was rooted using *L. roseus* KCTC 72321 as the outgroup.

To comparatively interpret the functional profile of KSO5, COG functional category analyses were conducted together with four representative *Acetobacter* genomes: *A. aceti* NBRC 14818, *A. cerevisiae* LMG 1625, *A. malorum* LMG 1746, and *A. pasteurianus* LMG 1262. Predicted protein sequences in FASTA amino acid format generated by Prokka were used as input for eggNOG-mapper. Because the eggNOG-mapper web server was unavailable during the analysis, annotation was performed locally using the following command:

emapper.py -i <input>.faa --output <input>_eggnog --data_dir /home/data1/biotools/eggnog_env/db/ --cpu 8

The COG_category field was extracted from each eggNOG-mapper output file. For proteins assigned to multiple COG categories, each single-letter category was counted independently. Unassigned entries and COG category S were combined into a single “unknown function” group for summary-level comparisons. To minimize bias caused by differences in genome size or the total number of annotated proteins, COG category counts were normalized to the total number of expanded COG assignments in each genome and expressed as percentages. The functional profile of KSO5 was then compared with each reference genome and with the mean percentage of the four non-KSO5 *Acetobacter* genomes. Differences were reported as percentage-point changes.

**Table 1:** Genome sequences used in the study.

No.	Name	Length (bp)	Accession No.
1	*Acetobacter cerevisiae* KSO5	3,257,599	CP172014
2	*A*. *pasteurianus* 386B	2,818,679	NC_021991.1
3	*A*. *pasteurianus* CICC 22518	2,772,347	NZ_CP39846.2
4	*A*. *pasteurianus* SRCM101468	2,996,610	NZ_CP021922.1
5	*A*. *pasteurianus* SRCM101342	2,754,755	NZ_CP021509.1
6	*A*. *pasteurianus* NBRC 101655	2,902,389	AP014881.1
7	*A. oryzifermentans* DM	3,127,455	NZ_CP022374.1
8	*A. oryzifermentans* SLV-7	2,799,488	NZ_CP011120.1
9	*A. ascendens* SRCM101447	2,901,846	NZ_CP021524.1
10	*A*. *persici* TMW2.1084	3,230,507	NZ_CP014687.1
11	*A*. *orientalis* FAN1	3,041,114	AP018515.1
12	*A*. *senegalensis* 108B	3,889,881	NZ_LN606600.1
13	*A*. *tropicalis* BDGP1	3,988,649	NZ_CP022699.1
14	*A*. *aceti* NBRC 14818^T^	3,596,270	NZ_AP023410.1
15	*A*. *aceti* JCM20276	3,743,357	NZ_AP023326.1
16	*A*. *aceti* TMW2.1153	3,725,037	NZ_CP014692.1
17	*Gluconacetobacter diazotrophicus* PA1 5	3,887,492	NC_011365.1
18	*Glu. diazotrophicus* PA1 5	3,944,163	NC_010125.1
19	*Komagataeibacter medellinensis* NBRC 3288	3,136,818	NC_016027.1
20	*Kom. xylinus* DSM 2325	3,353,346	NZ_CP025269.1
21	*Kom. xylinus* CGMCC 17276	3,527,401	NZ_CP041348.1
22	*Kom. xylinus* CGMCC 2955	3,563,314	CP024644.1
23	*Kom. xylinus* E25	3,447,725	CP004360.1
24	*Gluconobacter oxydans* 621H	2,704,625	NZ_LT900338.1
25	*G. oxydans* 621H	2,702,173	NC_006677.1
26	*A*. *pasteurianus* GHA7	2,927,634	CP157844
27	*A. syzygii* 9H-2	2,672,115	GCA_000964225
28	*A. pomorum* LHT 2458	3,308,689	GCA_002738225
29	*A. oryzoeni* B6^T^	3,153,180	GCF_004014775
30	*A. pomorum* DSM 11825	3,319,623	GCF_025995455.1
31	*A. ghanensis* LMG 23848	2,843,474	GCA_001499675
32	*A. pasteurianus* LMG 1262^T^	2,982,262	GCA_000285275
33	*A. pasteurianus* subsp. *paradoxus* LMG 1591	3,216,032	GCA_001766255
34	*A. pasteurianus* subsp. *ascendens* LMG 1590	2,999,217	GCA_001766235
35	*A. okinawensis* JCM 25146	3,166,244	GCA_000613865
36	*A. orleanensis* LMG 1583	3,007,844	GCF_001581005
37	*A. malorum* LMG 1746^T^	3,833,476	GCF_001580615
38	*A. cerevisiae* LMG 1625^T^	3,088,073	GCF_001580535
39	*A. vaccinii* KACC 21233	3,082,251	GCA_008365315

**Table 2:** Genome information for *A. cerevisiae* registered in NCBI.

No.	Strain Name	Source	Assembly	RefSeq	Level	Scaffolds
1	KSO5	Fruit vinegar	ASM4409463v1	GCF_44094635.1	Complete Genome	3
2	LMG 1625	Beer	ASM158053v1	GCF_001580535.1	Contig	157
3	DSM 14362	Tokyo University ^(1)^	ASM2599619v1	GCF_025996195.1	Contig	218
4	R-83281	Lambic beer	ASM2415826v1	GCF_024158265.1	Contig	128
5	R-82823	Lambic beer	ASM2415830v1	GCF_024158305.1	Contig	137
6	R-82821	Lambic beer	ASM2415828v1	GCF_024158285.1	Contig	144
7	R-82820	Lambic beer	ASM2415831	GCF_024158315.1	Contig	145
8	LMG 1545	Rice vinegar	ASM158110v1	GCF_001581105.1	Contig	108
9	LMG1608	Beer	ASM158107v1	GCF_001581075.1	Contig	177

^1^ This strain does not specify the isolation source and as DSM 14362 = LMG 1625 (type strain), analyses used LMG 1625.

### 2.7. Comparative Profiling of Mobile Genetic Element- and Plasmid-Associated Protein Modules in A. cerevisiae KSO5 and Related Strains

#### 2.7.1. Genome Quality Assessment and Normalization

Genome quality was assessed using assembly statistics and BUSCO-based completeness estimates (Supplementary Data S1). The evaluated metrics included genome size, number of contigs, N50, L50, GC content, assembly class, and BUSCO completeness. For KSO5, the strain-level genome assembly consisted of three replicons: contig 1, representing the complete circular chromosome, and contig 2 and contig 3, representing plasmids. These replicons were analyzed together as a single KSO5 genome assembly for quality assessment. In addition, sequence-level contamination screening was performed by examining ambiguous bases, gap characters, contamination-related FASTA header flags, and GC-content outliers. Genome assemblies deposited in NCBI were also regarded as having passed the standard NCBI submission validation process, including contamination screening.

#### 2.7.2. Identification and Quantification of Mobile Genetic Element-Associated Proteins

Candidate proteins associated with mobile genetic elements (MGEs) were identified from the KSO5 complete genome and seven comparator *A. cerevisiae* genomes using a predefined annotation-screening workflow. MGE-associated categories included transposase, integrase, phage-related proteins, recombinase, and repeat-protein annotations. Keyword searches were performed in a case-insensitive manner, and candidate hits were manually reviewed to remove obvious misannotations and to improve consistency across genomes.

When annotations overlapped between categories, proteins were assigned according to their primary functional annotation to avoid unintended double counting. The resulting counts were compiled for each chromosome- or genome-scale assembly. Because KSO5_P represents a discrete circular plasmid rather than a whole-genome assembly, it was recorded separately and excluded from formal chromosome/genome-scale rate comparisons.

#### 2.7.3. Normalized Comparison of MGE-Associated Annotation Counts

To support normalized comparisons beyond descriptive counts, MGE-associated annotation counts were analyzed using count-based generalized linear models. Poisson regression models were fitted with the logarithm of effective genome size as an offset term to estimate annotation rates per unit genome length. Rate ratios, 95% confidence intervals, and corresponding *p*-values were calculated for each MGE-associated category.

Because each genome represented a single assembly-level observation rather than replicated biological measurements, model-derived statistics were interpreted as exploratory genome-level annotation-rate comparisons rather than definitive biological inference. Overdispersion was assessed, and model outputs were interpreted conservatively when count distributions were sparse. Because the repeat-protein category included complete absence in KSO5 and may encompass heterogeneous repeat-domain proteins, this category was not used for strong statistical inference and was interpreted descriptively. All raw counts, normalization parameters, and statistical outputs are provided in Supplementary Data S2.

#### 2.7.4. Analysis of Plasmid-Associated Protein Modules

Plasmid-associated protein modules were identified based on annotations related to replication, partitioning, mobilization, stabilization, and toxin–antitoxin system components (Table 3). Representative proteins were assigned to functional modules, including RepA, RepB, RepC, MobA, MobC, and stabilization/toxin-associated proteins. Module assignment was based on protein product names, RefSeq accession information, and conserved-domain evidence, including COG, Pfam, and related domain annotations.

Comparative analysis of plasmid-associated modules across strains was performed qualitatively to assess module composition and structural diversity rather than by statistical testing, because plasmid architecture varies in organization and is not directly comparable as count-based features.

#### 2.7.5. Network Visualization of Plasmid-Associated Functional Modules in *A. cerevisiae* Strains

The plasmid-associated functional module network was visualized in Cytoscape v3.10.2 using the Prefuse Force Directed layout. Functional modules were identified based on RefSeq protein annotations and conserved-domain assignments from plasmid-associated proteins in each genome (Table 3). Node colors were assigned to differentiate strain identifiers from plasmid functional modules. The final layout was slightly adjusted manually to improve label visibility and high-resolution rendering, without altering node–edge relationships.

### 2.8. SNP Comparison of Protein-Coding Genes Involved in Acetic-Acid Resilience Mechanisms Across Eight A. cerevisiae Strains

Orthologous coding sequences (CDSs) were aligned across eight *A. cerevisiae* strains, using the KSO5 CDS as the reference sequence, and SNPs were called from the resulting multiple sequence alignments (MSAs). Variant and codon coordinates were lifted over from KSO5 to the type strain LMG1625 to ensure coordinate comparability. Alignment quality was assessed by BLAST-based checks including alignment length, mismatches, and gap openings, and SNP counts were verified to be consistent with BLAST mismatch counts.

To represent each acetic acid resilience related functional layer, one or more module-representative genes were selected per mechanism based on their functional relevance (Supplementary Table S1), reliable orthologous CDS alignment across the eight *A. cerevisiae* genomes, and suitability for codon-level SNP comparison. For enzymatic acetate metabolism (assimilation) related module, *acsA*, *ackA*, *pta*, *aarC*, *mqo*, and *acnA* were included. For efflux/transport, *oprM*_1 was selected. For chaperones/stress response, *groEL* and *dnaK* were included. For ROS detoxification, *srpA* was selected.

The module-representative gene panel was analyzed in depth by quantifying codon-level amino acid consequences, including synonymous, nonsynonymous, nonsense, and stop-loss variants, mapping variants onto conserved domains, and extracting key variants. In parallel, the same metrics were applied to non-module-representative genes in a genome-wide auxiliary screen to capture broader sequence-variation patterns.

For the acetate metabolism/assimilation-related module, *acsA*, *ackA*, *pta*, *aarC*, *mqo*, and *acnA* were selected because they represent acetate activation, acetate–acetyl phosphate–acetyl-CoA interconversion, CoA-transfer-linked acetate assimilation, and downstream central carbon metabolism. For the efflux/transport-related module, *oprM*_1 was selected because its encoded protein, OprM_1, represents an outer membrane efflux-associated component potentially involved in reducing intracellular stress caused by organic acids or other toxic compounds. For the chaperone/stress-response module, *groEL* and *dnaK* were selected because their encoded proteins, GroEL and DnaK, are representative molecular chaperones involved in proteome maintenance under environmental stress. For the ROS detoxification-related module, *srpA* was selected because its encoded protein, SrpA, is a catalase-related peroxidase representing oxidative-stress defense. The analysis was therefore designed as an exploratory, mechanism-oriented comparison of sequence variation among representative genes, rather than as a direct functional validation of individual acid-tolerance determinants.

## 3. Results and Discussion

### 3.1. Morphology and Physiology of A. cerevisiae KSO5

**Figure 1:**
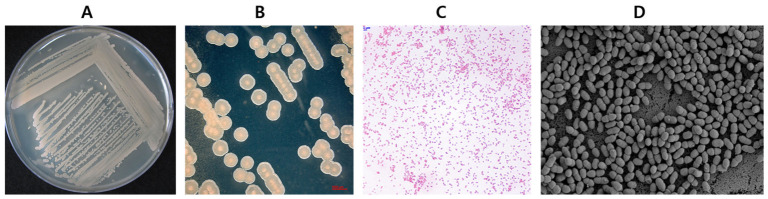
Morphology of KSO5 strain. (**A**) Colonies grown on YGC agar medium. (**B**) Colonies observed under a stereomicroscope. (**C**) Gram-stained cells. (**D**) Scanning electron microscopy (SEM) images.

An acetic acid bacteria (AAB) strain, *A. cerevisiae* KSO5, was isolated from farm-produced omija (magnolia berry, *Schisandra chinensis*) fruit vinegar. The general characteristics of the strain are shown in Figure 1. Colonies grown on YGC solid medium were circular, convex, opaque, and cream-colored (Figure 1A,B), consistent with typical *Acetobacter* morphology. The cells were confirmed as Gram-negative (Figure 1C). Scanning electron microscopy (SEM) revealed that the cells were rod-shaped with oval ends, measuring 0.3–0.4 µm in diameter and 0.9–1.2 µm in length. The cells typically appeared in pairs, with some single cells observed (Figure 1D). No flagella, stalks, or prosthecae were observed.

**Figure 2:**
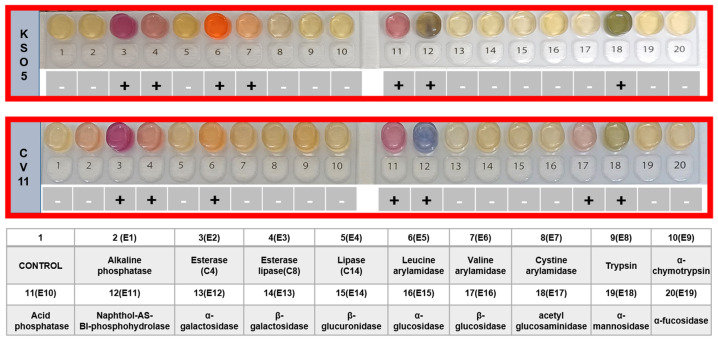
Enzyme activity of KSO5 determined using the API ZYM kit. Enzyme activities were assessed based on the hydrolysis of 19 substrates, following the interpretation criteria provided by the API manufacturer (bioMérieux, Marcy-l’Étoile, France). Symbol: +, positive; -, negative; CV11, *A. malorum* CV11 (KACC 92076P).

The KSO5 strain exhibited positive enzymatic activities for esterase (C4), esterase lipase (C8), leucine arylamidase, valine arylamidase, acid phosphatase, naphthol-AS-BI-phosphohydrolase, and acetyl-glucosaminidase (Figure 2). When compared to the closely related *A. malorum* CV11 (KACC 92076P), KSO5 showed positive valine arylamidase activity, whereas β-glucosidase activity was undetectable. Minor variations were observed in specific enzymatic activities, but overall, the enzymatic profiles of the KSO5 and CV11 strains were largely similar. The KSO5 strain has been deposited in the Korean Agricultural Culture Collection (KACC 92352P), part of the National Institute of Agricultural Sciences.

These morphological and physiological characteristics support the classification of KSO5 as an acetic acid bacterium associated with vinegar-fermentation and provide a phenotypic foundation for subsequent genome-level and comparative analyses.

### 3.2. Genome Features of A. cerevisiae KSO5

**Figure 3:**
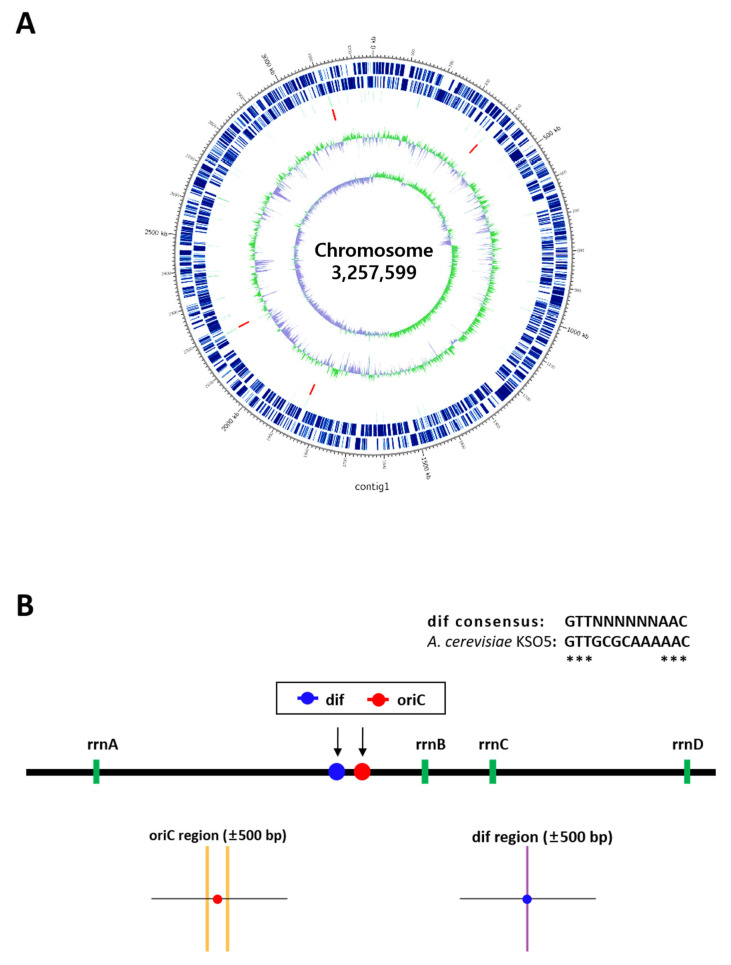
Features of the KSO5 chromosome. (**A**) Circular map (outer → inner): coordinates; CDS +/−; tRNA; rRNA; GC content; GC skew. (**B**) Linearised genome (3,257,599 bp) highlighting oriC and dif with rrn operons; insets magnify ±500 bp motifs (orange for DnaA-box, purple for dif site). Asterisks denote conserved positions matching the Alphaproteobacteria consensus dif sequence, 5′-GTTN{6}AAC-3′.

The complete genome of strain KSO5 was determined and deposited in NCBI GenBank (accession no. CP172014–CP172016). It comprises one circular chromosome (3,257,599 bp; GC content, 57.8%; 2889 protein-coding sequences [CDSs]; 54 tRNA genes; and 12 rRNA genes arranged in four rRNA operons) and two circular plasmids (KSO5_P1, 4905 bp, 56.2% GC, 5 CDSs; and KSO5_P2, 4820 bp, 56.1% GC, 4 CDSs) (Figure 3A; Table 4). In total, the genome spans 3,267,324 bp with an overall GC content of 57.8% and encodes 2898 protein-coding genes, in addition to the 54 tRNA genes and 12 rRNA genes noted above (Table 4). The genomic GC content falls within the range reported for members of the genus *Acetobacter*.

The origin of chromosomal replication (oriC) in KSO5 was predicted at position 1,582,749 bp, based on the coincidence of the GC skew maximum [13,14], clustering of at least seven *Alphaproteobacteria*-type DnaA-box motifs (TTATCCACA and variants) [15,16], and the presence of an AT-rich region adjacent to replication initiation genes (*dnaA*, *dnaN*, *recF*, *gyrB*) [15,17]. The putative replication termination site (dif) was identified at position 1,531,352 bp, showing perfect alignment to the *Alphaproteobacteria* consensus dif sequence (5′-GTTN{6}AAC-3′) and proximity to the *xerC*/*xerD* recombinase genes [16]. These loci were further validated by cumulative GC skew analysis, revealing the oriC and dif positions at the skew maximum and minimum, respectively [13,14]. Four rrn operons were mapped at positions: rrnA, 385,463–390,694 bp; rrnB, 1,848,948–1,854,179 bp; rrnC, 2,189,505–2,194,736 bp; rrnD, 3,114,803–3,120,034 bp [17] (Figure 3B).

The complete genome structure of KSO5, comprising a circular chromosome and plasmid components, provided a high-confidence genomic framework for downstream functional annotation, comparative genomics, and stress-resilience analysis.

### 3.3. Comparative Genomics and Phylogenomic Placement of A. cerevisiae KSO5

To determine the taxonomic placement of strain KSO5, its 16S rRNA gene sequence was first compared with those of representative *Acetobacter* strains (Supplementary Figure S1). Strain KSO5 showed the highest sequence similarity to *A. cerevisiae* LMG 1625^T^ at 99.71%, and its 16S rRNA gene sequence was deposited in the NCBI GenBank database under accession number PP478110.

In the 16S rRNA gene-based phylogenetic analysis using sequences from the 39 genomes listed in Table 1, strain KSO5 was placed within the genus *Acetobacter* in the neighbor-joining tree and was positioned close to *A. cerevisiae* LMG 1625 ^T^, supporting its assignment to the *A. cerevisiae* lineage (Supplementary Figure S2).

**Figure 4:**
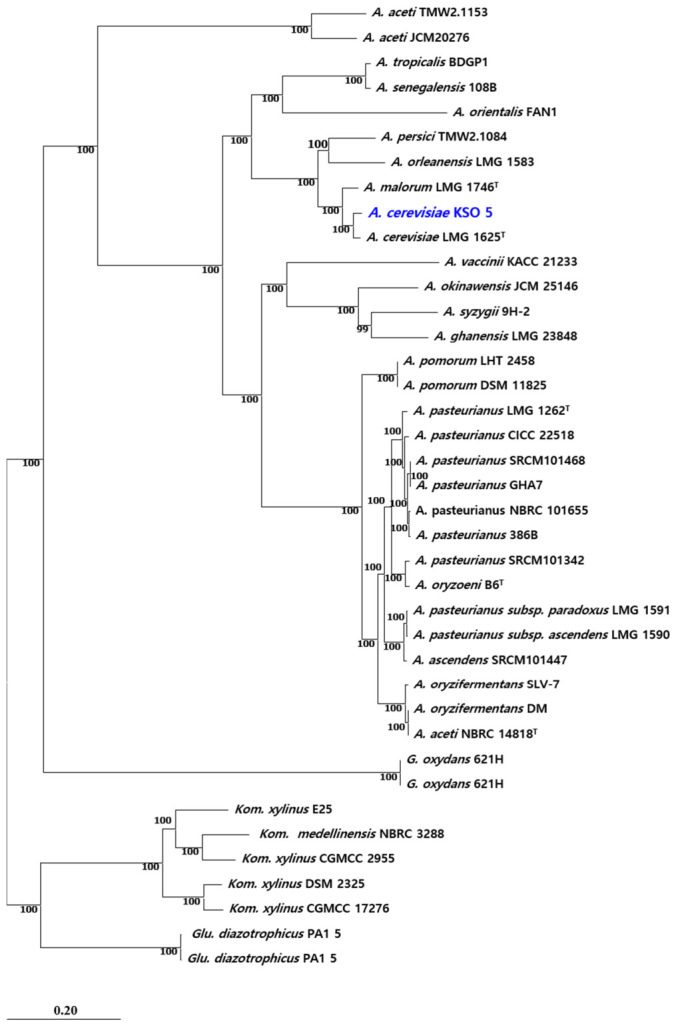
Maximum-likelihood phylogenetic tree based on the concatenated alignment of 628 core genes identified by Roary from 39 acetic acid bacterial genomes (Table 1). The tree was inferred using IQ-TREE with automatic model selection and 1000 ultrafast bootstrap replicates. Bootstrap values are shown at the nodes. The scale bar indicates the number of substitutions per site. *Lichenicoccus roseus* KCTC 72321 was used as the outgroup for rooting but is not shown in the final tree for clarity.

To further clarify the phylogenomic position of KSO5, a sequence-based core genome phylogeny was constructed. Roary identified 25,103 total genes across the analyzed genome set, including 628 core genes, 262 soft-core genes, 3519 shell genes, and 20,694 cloud genes. The 628 core genes were concatenated and used to infer a maximum-likelihood phylogenetic tree using IQ-TREE. The resulting core genome-based tree exhibited a well-resolved topology with high bootstrap support for most major nodes. Strain KSO5 clustered with *A. cerevisiae* LMG 1625 ^T^ with strong bootstrap support, confirming its placement within the *A. cerevisiae* lineage (Figure 4). This placement was consistent with the GTDB-based phylogenomic tree constructed using conserved marker proteins (Supplementary Figure S3).

The close relationship between KSO5 and *A. cerevisiae* LMG 1625^T^ was further supported by genome-wide similarity indices. KSO5 showed 97% average nucleotide identity (ANI) with *A. cerevisiae* LMG 1625 ^T^, exceeding the commonly accepted species delineation threshold of 95–96% (Supplementary Data S3), whereas its ANI value with *A. malorum* LMG 1746 was 93%, below the species boundary. Similarly, digital DNA–DNA hybridization (dDDH) between KSO5 and *A. cerevisiae* LMG 1625^T^ was 71%, meeting the ≥70% species delineation criterion (Supplementary Data S4).

Taken together, the 16S rRNA gene sequence comparison, 16S rRNA gene-based phylogeny, core genome-based phylogeny, ANI, and dDDH analyses consistently support the taxonomic assignment of strain KSO5 to *Acetobacter cerevisiae*. In particular, the core genome-based phylogeny provided a robust phylogenomic framework for resolving the evolutionary relationship of KSO5 within closely related *Acetobacter* taxa. This genome-level confirmation is important because subsequent comparative and functional analyses were conducted within a species-level framework rather than relying solely on single-gene-based identification.

### 3.4. Comparative COG Functional Profile of A. cerevisiae KSO5

**Figure 5:**
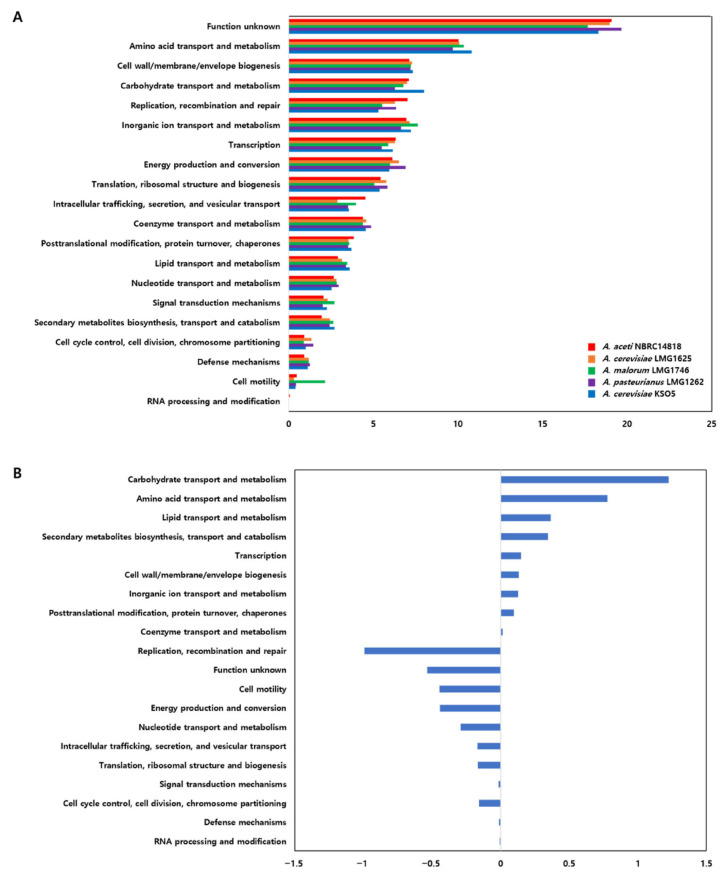
Comparative COG functional category distribution of *A. cerevisiae* KSO5 and related *Acetobacter* genomes. COG categories were assigned using eggNOG-mapper based on Prokka-predicted protein sequences. Multi-category COG assignments were expanded into single-letter categories, and counts were normalized to the total number of expanded COG assignments in each genome. (**A**) The bar plot shows the percentage distribution of COG functional categories among *A. aceti* NBRC 14818, *A. cerevisiae* LMG 1625, *A. malorum* LMG 1746, *A. pasteurianus* LMG 1262, and *A. cerevisiae* KSO5. (**B**) The difference plot shows the percentage-point difference between KSO5 and the mean of the four comparator genomes. Positive values indicate categories relatively enriched in KSO5, whereas negative values indicate categories relatively reduced in KSO5.

The comparative COG analysis compared the KSO5 genome with those of related *Acetobacter* genomes. The overall COG distribution of KSO5 was similar to those of *A. aceti* NBRC 14818, *A. cerevisiae* LMG 1625, *A. malorum* LMG 1746, and *A. pasteurianus* LMG 1262, suggesting that KSO5 retains the conserved functional organization typical of the genus. However, normalized comparison identified several meaningful differences (Figure 5).

The COG category dataset contained 3281 category assignments for *A. cerevisiae* KSO5, which fell within the range observed for the comparator *Acetobacter* genomes, 3011–3632 assignments. In all genomes, the largest category corresponded to function unknown or poorly characterized proteins, accounting for 17.68–19.66% of total COG assignments. In KSO5, this category represented 18.32%, slightly lower than the comparator mean of 18.85% (Figure 5A).

The most prominent difference was the higher relative proportion of genes assigned to carbohydrate transport and metabolism, category G, in KSO5 compared with the comparator average, 8.02% versus 6.79%, corresponding to a +1.23 percentage-point difference. KSO5 also showed a higher proportion of amino acid transport and metabolism, category E, 10.82% versus 10.04%, +0.78 percentage points. These patterns were maintained when KSO5 was compared specifically with the species-level reference strain *A. cerevisiae* LMG 1625; KSO5 showed higher proportions of category G, 8.02% versus 7.00%, and category E, 10.82% versus 10.08%. Therefore, the enrichment of carbohydrate- and amino acid-related COG categories in KSO5 may represent a strain-level expansion of nutrient acquisition and metabolic utilization potential within the conserved *Acetobacter* genomic background. KSO5 also showed modestly higher proportions of lipid transport and metabolism, category I, 3.60% versus 3.23%, and secondary metabolite biosynthesis, transport, and catabolism, category Q, 2.71% versus 2.37%, compared with the comparator average (Figure 5B).

In this context, genome-encoded proteins related to carbohydrate oxidation indicate that KSO5 has a versatile genetic capacity for carbohydrate utilization (Supplementary Table S3; Figure 5B). The pentose phosphate pathway (PPP) is strongly represented by G6PDH and 6PGD, which is consistent with the potential production of NADPH and ribulose-5-phosphate for anabolism and redox homeostasis [18,19,20]. KSO5 also encodes EMP-associated enzymes, suggesting a possible capacity to reroute fructose-6-phosphate and triose phosphates as fermentation conditions shift. Periplasmic PQQ-dependent glucose dehydrogenase may accelerate extracellular glucose oxidation and contribute to medium acidification, providing a potential niche advantage. Additional polyol and sugar-acid routes, including FAD-dependent sorbitol dehydrogenase (D-sorbitol to L-sorbose to D-fructose), mannitol oxidation via polyol oxidoreductase, and glycerol utilization via GlpF/GlpK and FAD/NAD-linked G3PDHs, may feed EMP intermediates and increase carbon-processing potential [21,22,23]. Osmotolerance and envelope resilience are supported by trehalose metabolism (OtsA/OtsB/TreA) [24] and large-conductance mechanosensitive channels (MscL/MscS/MscK) [25]. In addition, abundant transporters, including approximately 50 ABC transporters and multiple symporters/permeases, suggest broad uptake potential for sugars, polyols, organic acids, and ions, thereby supporting central carbon metabolism and respiratory activity.

**Figure 6:**
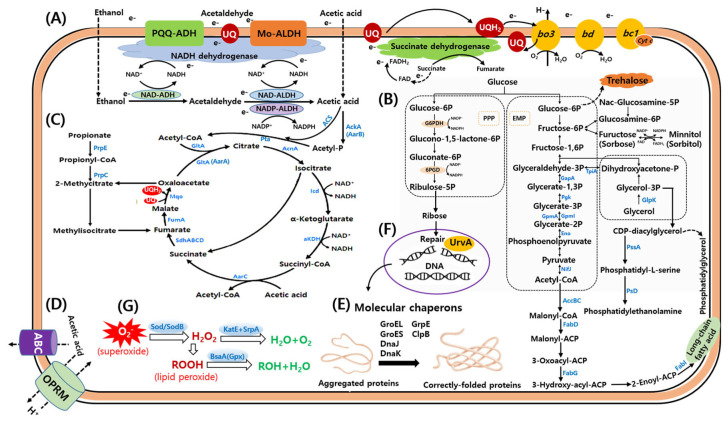
CoSchematic illustration of the key metabolic pathways and regulatory networks associated with the expressed proteins. The proposed mechanisms include: (**A**) membrane-bound dehydrogenases and the respiratory chain; (**B**) carbohydrate oxidation; (**C**) intracellular acetate activation and assimilation; (**D**) acetic acid extrusion; (**E**,**F**) stress response systems, including molecular chaperone-mediated protein folding and DNA repair; and (**G**) toxin inactivation and persistence-related stress response. Detailed protein annotation data are provided in Supplementary Tables S1–S3.

These respiratory and metabolic layers may integrate with three genome-encoded acetate-handling strategies that mitigate intracellular acid stress (Supplementary Tables S1 and S2): (i) putative PMF-driven and ABC-type transport systems that may contribute to membrane transport and acid-stress adaptation (Figure 6D), although their substrate specificity requires further validation; (ii) conversion of acetate to acetyl-CoA via acetate kinase/phosphotransacetylase/acetyl-CoA synthetase, thereby channeling acetate into central metabolism; and (iii) a specialized TCA branch involving succinyl-CoA:acetate CoA-transferase (AarC), which has previously been associated with secondary growth and acetic-acid resistance in AAB (Figure 6C). In addition, membrane homeostasis-related proteins, including ACC and FabD/FabG/FabI, together with proteins involved in phosphatidylserine and phosphatidylethanolamine biosynthesis and N-acetylglucosaminidase proteins, further support the genome-wide evidence for enhanced membrane-associated adaptation, transport/catabolic versatility, and metabolic flexibility in KSO5 (Supplementary Table S3; Figure 6B). This interpretation is consistent with the modestly higher proportions of lipid transport and metabolism (COG category I) and secondary metabolite biosynthesis, transport, and catabolism (COG category Q) in KSO5 compared with the comparator average [26].

In contrast, KSO5 had a lower relative proportion of genes assigned to replication, recombination, and repair, category L, than the comparator genomes, 5.30% versus 6.30%, −0.99 percentage points (Figure 5B). KSO5 also showed lower representation of energy production and conversion, category C, 5.94% versus 6.39%, −0.44 percentage points, and cell motility, category N, 0.40% versus 0.84%, −0.45 percentage points. The lower cell motility value was mainly influenced by the high proportion of this category in *A. malorum* LMG 1746, whereas KSO5 was similar to the other comparator genomes. Therefore, these lower normalized COG proportions should be interpreted cautiously and should not be taken to indicate the absence of the corresponding core functions.

Overall, the comparative COG profile (Figure 5) and genome-encoded protein repertoire (Supplementary Tables S1–S3) suggest that KSO5 has an expanded genetic potential for carbohydrate utilization, redox balancing, membrane transport, and stress adaptation within a conserved *Acetobacter* genomic background. Although the relative proportion of category C was slightly lower in KSO5 than in the comparator genomes, the presence of carbohydrate oxidation enzymes, central metabolic pathway genes, and transporter systems suggests that KSO5 retains sufficient respiratory and redox-related capacity to support oxidative metabolism. Likewise, the lower relative proportion of category L should be interpreted as a genome-wide normalized pattern rather than the absence of recombination- or mobile-element-associated proteins. These features are consistent with potential metabolic flexibility under fermentation-associated acidic and osmotic stress conditions; however, transcriptomic, proteomic, or physiological validation would be required to confirm their functional activity.

### 3.5. Comparative Ethanol-Stress Phenotyping of A. cerevisiae KSO5 and Reference Strains

To evaluate the physiological robustness of *A. cerevisiae* KSO5 under ethanol-stress conditions, its growth, acidification capacity, and alcohol consumption profile were compared with those of reference strains during 10 days of cultivation. Overall, KSO5 exhibited a balanced ethanol-stress phenotype characterized by stable growth, sustained acid production, and rapid alcohol consumption, whereas the reference strains showed more condition-dependent responses.

#### 3.5.1. Growth Profiles Under Different Ethanol Concentrations

**Figure 7:**
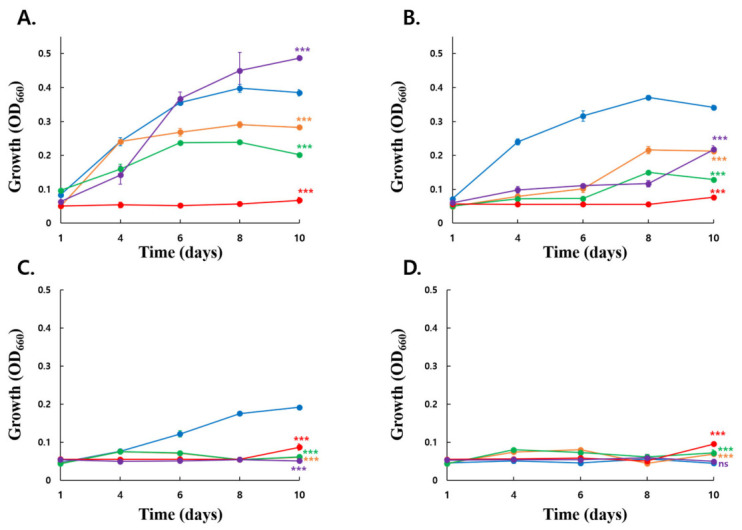
Time-course growth profiles of KSO5 and type strains under different ethanol concentrations. Growth (OD_660_) of five strains, *A. aceti* NBRC 14818 (red filled circle), *A. cerevisiae* LMG 1625 (orange filled circle), *A. malorum* LMG 1746 (green filled circle), *A. pasteurianus* LMG 1262 (purple filled circle), and *A. cerevisiae* KSO5 (blue filled circle), was monitored during cultivation in media containing (**A**) 5%, (**B**) 7%, (**C**) 9%, or (**D**) 10% ethanol. Data are presented as mean ± SD from four biological replicates (n = 4). Symbols adjacent to the day 10 data points indicate significant differences compared with KSO5 within each ethanol concentration, as determined by one-way ANOVA followed by Dunnett’s multiple comparison test. ns, not significant; *** *p* < 0.001.

Growth profiles differed significantly among strains under all tested ethanol concentrations (Figure 7). At 5% ethanol, KSO5 showed higher growth than *A. cerevisiae* LMG 1625, *A. malorum* LMG 1746, and *A. aceti* NBRC14818 by day 10, whereas *A. pasteurianus* LMG 1262 exhibited a higher final growth level than KSO5. Under 7% and 9% ethanol conditions, KSO5 displayed the strongest growth performance and maintained significantly higher OD_660_ values than all tested type strains at day 10 (Dunnett’s test, *p* < 0.001). In contrast, growth was markedly suppressed in all strains at 10% ethanol. Under this condition, KSO5 no longer showed a growth advantage and exhibited significantly lower growth than LMG 162*5*, LMG 1746, and NBRC14818, while no significant difference was observed relative to LMG 1262. These results indicate that KSO5 possesses superior growth fitness under moderate ethanol stress, particularly at 7–9% ethanol, but that this ada most severe condition tested.

#### 3.5.2. Acid Production Under Ethanol-Stress Conditions

**Figure 8:**
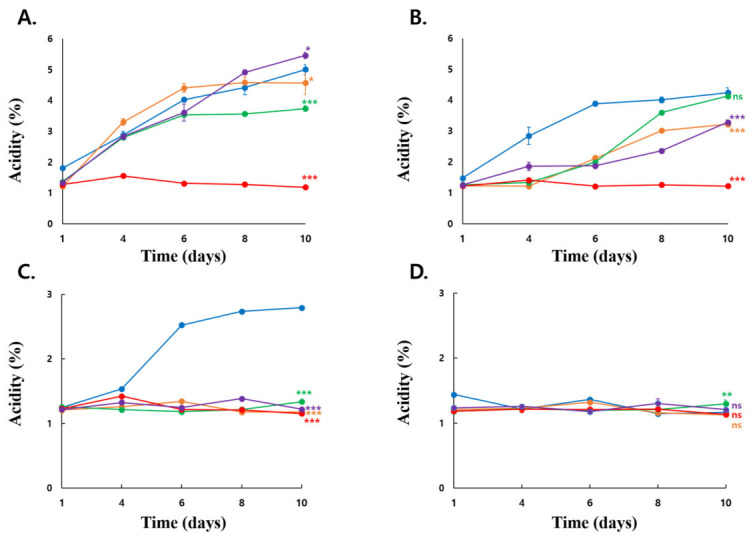
Time-course acidity production by KSO5 and type strains under different ethanol concentrations. Acidity production (%) of five strains, *A. aceti* NBRC 14818 (red filled circle), *A. cerevisiae* LMG 1625 (orange filled circle), *A. malorum* LMG 1746 (green filled circle), *A. pasteurianus* LMG 1262 (purple filled circle), and *A. cerevisiae* KSO5 (blue filled circle), was measured during cultivation in media containing (**A**) 5%, (**B**) 7%, (**C**) 9%, or (**D**) 10% ethanol. Data are presented as mean ± SD from four biological replicates (n = 4). Symbols adjacent to the day 10 data points indicate significant differences compared with KSO5 within each ethanol concentration, as determined by one-way ANOVA followed by Dunnett’s multiple comparison test. ns, not significant; * *p* < 0.05; ** *p* < 0.01; *** *p* < 0.001.

Acidity production also differed significantly among strains across the tested ethanol concentrations (Figure 8). At 5% ethanol, KSO5 produced more acidity than LMG 1625, LMG 1746, and NBRC14818, but less acidity than LMG 1262 at day 10. At 7% ethanol, KSO5 showed the highest acidity production and was significantly higher than LMG 1625, NBRC14818, and LMG 1262, whereas no significant difference was observed between KSO5 and LMG 1746. Notably, at 9% ethanol, KSO5 exhibited the strongest acidity production throughout the later cultivation period and remained significantly higher than all type strains at day 10 (Dunnett’s test, *p* < 0.001). At 10% ethanol, acidity production was substantially reduced in all strains, and only LMG 1746 showed a significantly higher value than KSO5 at day 10. Overall, these results demonstrate that KSO5 has a particularly strong acid-producing capacity under 7–9% ethanol, with the most pronounced advantage observed at 9% ethanol.

#### 3.5.3. Alcohol Consumption Capacity Under Ethanol-Stress Conditions

**Figure 9:**
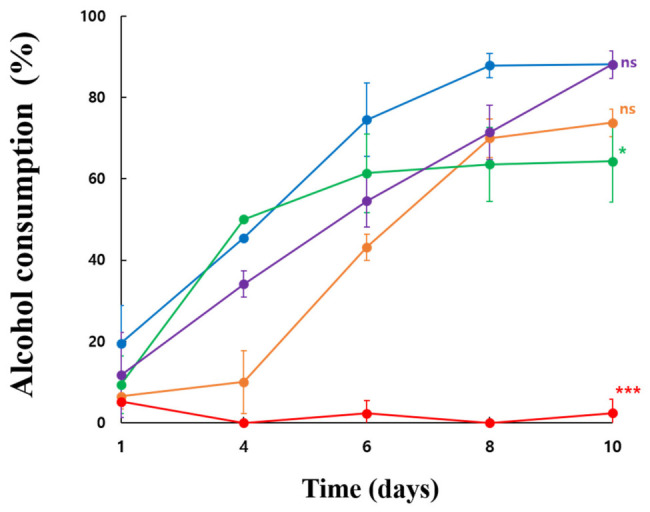
Time-course ethanol consumption by KSO5 and type strains in 5% ethanol medium. Ethanol consumption (%) of five strains, *A. aceti* NBRC 14818 (red filled circle), *A. cerevisiae* LMG 1625 (orange filled circle), *A. malorum* LMG 1746 (green filled circle), *A. pasteurianus* LMG 1262 (purple filled circle), and *A. cerevisiae* KSO5 (blue filled circle), was monitored during cultivation in 5% ethanol medium. Ethanol consumption was calculated based on the decrease in residual ethanol relative to the initial ethanol concentration. Data are presented as mean ± SD from two biological replicates (n = 4). Symbols adjacent to the day 10 data points indicate significant differences compared with KSO5, as determined by one-way ANOVA followed by Dunnett’s multiple comparison test. ns, not significant; * *p* < 0.05; *** *p* < 0.001.

In 5% ethanol medium, ethanol consumption increased over time in all strains, but the extent of ethanol consumption differed among strains (Figure 9). KSO5 and LMG 1262 showed the highest ethanol consumption by day 10, whereas NBRC14818 displayed the lowest consumption. Compared with KSO5, NBRC14818 showed significantly lower ethanol consumption at day 10 (Dunnett’s test, *p* < 0.001), and LMG 1746 also showed a lower value (*p* < 0.05). In contrast, differences between KSO5 and LMG 1625 or between KSO5 and LMG 1262 were not statistically significant at day 10. Although the alcohol-consumption experiment was based on two biological replicates, the results consistently suggest that KSO5 has a high ethanol-oxidizing capacity comparable to that of LMG 1262 and superior to that of most of the other reference strains tested.

The alcohol consumption profile was generally consistent with the acidity data. Strains with higher alcohol consumption tended to show greater acid accumulation, whereas the strain with negligible alcohol consumption also showed little acid production. Overall, KSO5 displayed a desirable ethanol-stress phenotype characterized by early alcohol consumption, sustained acid production, and stable growth. Compared with the reference strains, KSO5 appears to be particularly advantageous under ethanol-rich conditions where rapid ethanol conversion and acidification are required. These findings support the potential application of KSO5 as a functional starter or industrial strain for fermentation processes involving ethanol stress.

The phenotypic differences observed under ethanol-stress conditions could be further contextualized by the functional annotation profiles of the tested strains (Figure 5). Although the overall COG distribution was broadly comparable among the strains, *A. cerevisiae* KSO5 showed relatively higher or comparable proportions of genes assigned to carbohydrate transport and metabolism, amino acid transport and metabolism, inorganic ion transport and metabolism, lipid transport and metabolism, and secondary metabolite transport and catabolism (Figure 5B). These functional categories do not directly prove ethanol tolerance; however, they may support metabolic flexibility, ion homeostasis, membrane adaptation, and stress-associated substrate utilization under ethanol-rich conditions.

In contrast, *A. aceti* NBRC14818 showed negligible growth, acid production, and alcohol consumption even at 5% ethanol, despite the general recognition of *A. aceti* NBRC14818 as an ethanol-associated acetic acid bacterium in wine environments [27,28]. This discrepancy indicates that ethanol tolerance and ethanol-to-acid conversion are not determined solely by species identity or type-strain status, but rather by strain-specific functional capacity under defined culture conditions [29,30]. In AAB, ethanol oxidation requires coordinated activity of membrane-associated dehydrogenases, cofactor-dependent redox reactions, respiratory energy generation, membrane integrity, and acid-stress adaptation [3,22,31]. Therefore, the poor performance of *A. aceti* NBRC14818 suggests that the presence of broad metabolic categories alone was insufficient to sustain active ethanol oxidation under the present experimental conditions.

Notably, *A. pasteurianus* LMG1262 and KSO5 retained stronger physiological activity than *A. aceti* NBRC14818, although their performance patterns differed depending on ethanol concentration. *A. pasteurianus* showed a strong response particularly under lower ethanol-stress conditions, whereas KSO5 maintained growth, acidification, and alcohol consumption more consistently across increasing ethanol concentrations. These findings suggest that KSO5 possesses an integrated ethanol-stress phenotype involving both cellular robustness and functional ethanol conversion. Thus, the combined phenotypic and functional annotation results support the conclusion that KSO5 is better adapted than the *A. aceti* type strain to ethanol-rich fermentation environments requiring sustained growth, acid production, and alcohol utilization.

### 3.6. Comparative Phylogenomic Analysis of A. cerevisiae KSO5

**Figure 10:**
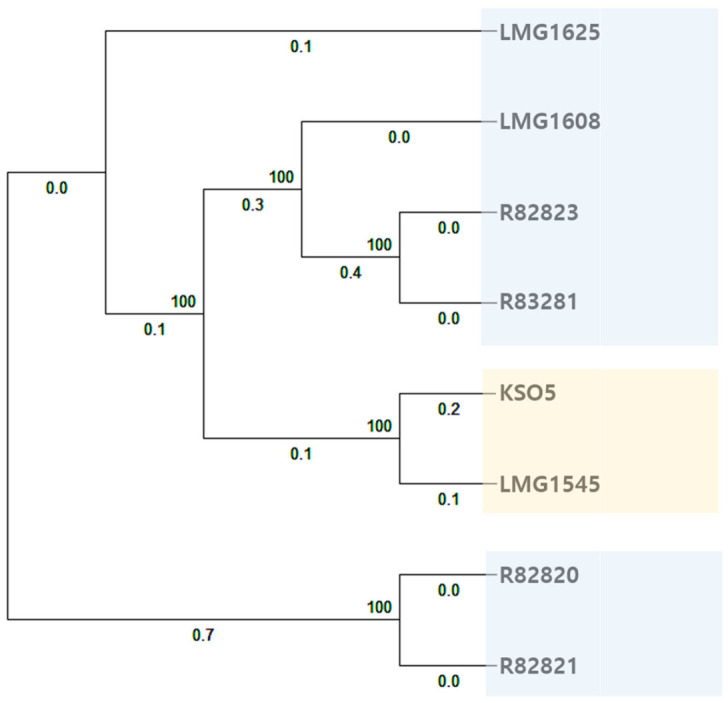
Phylogeny of eight *A. cerevisiae* strains from accessory gene presence–absence. Terminal labels are strain names. Light-blue shading marks strains isolated from beer (LMG1625, LMG1608, R82823, R83281, R82820, and R82821), and pale-yellow shading marks the two vinegar-derived strains—KSO5 from fruit vinegar and LMG1545 from cereal vinegar.

Across eight *A. cerevisiae* genomes, the pan-genome comprised 2012 core, 2024 shell, and 1572 cloud genes. An accessory gene presence–absence phylogeny was inferred from the binary matrix of all non-core gene clusters (shell and cloud genes) across the eight genomes (Figure 10), thereby reflecting overall genome-content similarity rather than sequence divergence at conserved loci. In this genome-content framework, KSO5 clustered as a sister strain to LMG 1545, whereas the remaining strains formed two additional clades (R82820/21 and R82823/83281), with LMG 1608 and LMG 1625 branching within the broader set of beer-associated isolates. Notably, the KSO5–LMG 1545 grouping corresponded to the two vinegar-derived strains, while most beer-associated strains grouped outside this clade. However, because the current dataset was limited to eight genomes and did not include systematic phenotyping, this pattern was reported as a difference in accessory gene repertoires rather than as evidence of predictable, mechanism-specific ecological differentiation. Consistent with accessory-genome turnover, the strain set showed variation in mobile genetic element-associated functions (e.g., transposases, integrases, and phage-related genes), which was consistent with differences in the variable gene repertoire captured by the genome-content analysis.

### 3.7. Comparative Profiling of Mobile Genetic Elements and Plasmid-Associated Protein Modules with Genetic Implications in A. cerevisiae KSO5 and Related Strains

**Figure 11:**
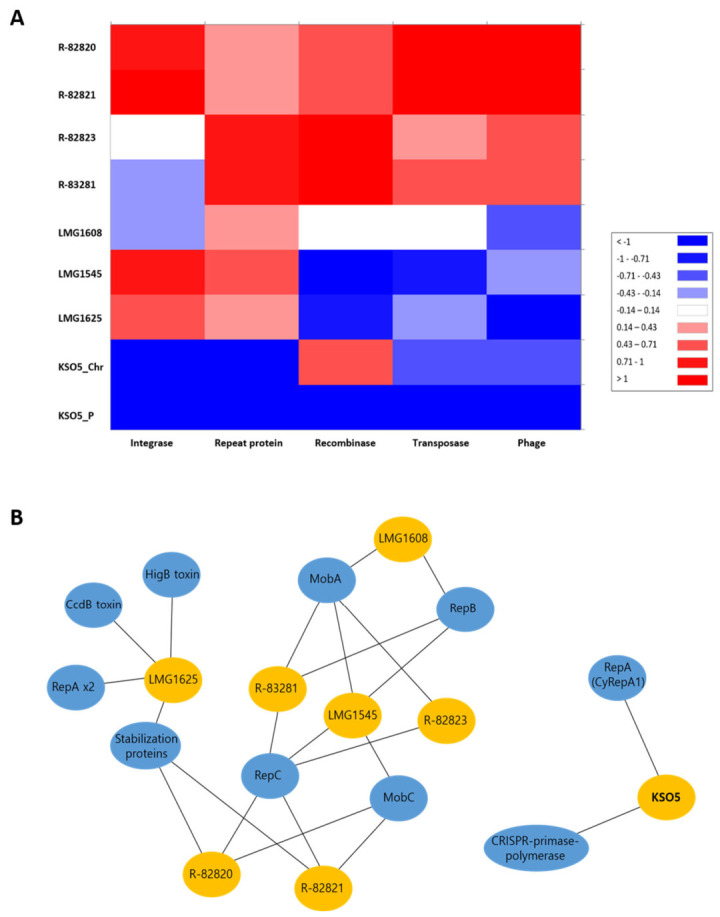
Comparative profiling of mobile genetic elements (MGEs) and plasmid-associated protein modules in *Acetobacter cerevisiae* KSO5 and related strains. (**A**) The heatmap shows the distribution of transposase, integrase, phage-related, recombinase, and repeat-protein annotations across KSO5_Chr, KSO5_P, and seven related *A. cerevisiae* strains. Warmer colors indicate higher annotation counts, whereas cooler colors indicate lower counts. KSO5_P was treated as a plasmid-level descriptive case and excluded from the chromosome/genome-scale rate-based comparison. (**B**) Blue nodes represent *A. cerevisiae* strains, and orange nodes indicate plasmid-associated functional modules identified by genome annotation. Edges indicate the presence of each module in the corresponding strain. KSO5 carries a CyRepA1-family replication protein with a CRISPR-associated primase–polymerase, whereas the related strains show alternative plasmid module organizations, including RepA-associated stabilization/toxin–antitoxin components and RepB/RepC/Mob-type architectures.

To further interpret the genome-content structure shown in Figure 10, we compared mobile genetic element (MGE)-associated annotation profiles across the eight *A. cerevisiae* strains and analyzed plasmid-associated protein modules separately (Supplementary Data S1 and S2). Descriptively, the KSO5 chromosome contained 18 transposases, 2 integrases, 7 phage-related genes, and 8 recombinases, whereas KSO5_P contained only 6 transposases and 1 recombinase and lacked integrase, phage-related, and repeat-protein annotations. Because KSO5_P represents a discrete circular plasmid, formal between-strain statistical comparison was restricted to chromosome/genome-scale assemblies (Figure 11A).

When modeled as count rates normalized to effective genome size, the KSO5 chromosome showed lower transposase and phage-related annotation densities than the seven comparator genomes (transposase: rate ratio = 0.61, 95% CI 0.38–0.99, *p* = 0.043; phage-related genes: rate ratio = 0.40, 95% CI 0.19–0.85, *p* = 0.018). Integrase-associated annotations also tended to be lower in KSO5, although the difference did not reach statistical significance (rate ratio = 0.31, 95% CI 0.08–1.28, *p* = 0.106). In contrast, recombinase abundance was not reduced in KSO5 (rate ratio = 1.14, 95% CI 0.54–2.41, *p* = 0.726). No repeat-protein annotation was detected in KSO5; however, this category was interpreted descriptively rather than as formal evidence for genome-wide repeat depletion, because repeat-domain annotations may be functionally heterogeneous. Overall, these data support a selective reduction in several chromosome-scale MGE-associated annotation categories in KSO5 rather than a uniform reduction across all categories.

At the plasmid-module level, KSO5 encoded a CyRepA1-family replication protein together with a CRISPR-associated primase-polymerase, whereas the comparator strains displayed alternative organizations, including RepA-associated stabilization/toxin-antitoxin components in LMG1625, RepB/RepC/Mob-type architectures in LMG1545, R-82823, and R-83281, a reduced RepC + MobC-type organization in R-82820 and R-82821, and a lean RepB + MobA configuration in LMG1608 (Figure 11B and Table 3). These comparisons indicate that the KSO5 plasmid has a compact and atypical module organization relative to the related strains, although the functional implications remain inferential and require experimental validation.

### 3.8. Genetic Architecture of Acetic-Acid Resilience Across A. cerevisiae KSO5

AAB routinely face high organic-acid loads, especially acetic acid, which sharply depresses growth and metabolism [32]. To interpret the phenotypic performance of KSO5 under ethanol- and acid-associated stress, we further examined genomic modules related to acetate metabolism, acid efflux, molecular chaperones, oxidative-stress detoxification, and sequence variation in representative resilience-associated proteins. These analyses suggest that KSO5 (i) leverages enzymatic acetate metabolism (assimilation), (ii) accelerates export through membrane transporters, including ABC systems and proton-motive-force-driven pumps, (iii) preserves proteome integrity by inducing stress chaperones such as DnaK and GroEL, and (iv) mitigates oxidative damage by detoxifying reactive oxygen species through enzymes such as superoxide dismutase, catalase, and peroxidases [33]. Consistent with this framework, the circular genome of *A. cerevisiae* KSO5 encodes extensive complements across all four layers, indicating a robust, genome-encoded capacity for acid tolerance [1,34] (Figure 6).

#### 3.8.1. Enzymatic Acetate Metabolism (Assimilation)

KSO5 harbored a respiratory and redox architecture compatible with rapid periplasmic oxidative fermentation under acid stress, including multiple PQQ-dependent alcohol and molybdopterin aldehyde dehydrogenase systems (PQQ-ADH/Mo-ALDH) that may help decouple carbon oxidation from cytosolic NAD(H) balance (Figure 6A; Supplementary Table S2). Notably, the membrane-bound PQQ-ADH module appeared to comprise the major AdhA/AdhB components without a clearly identifiable AdhS-like small subunit. This configuration resembled the two-subunit (AdhA/AdhB) membrane-bound ADH system reported in *Komagataeibacter* and *Gluconacetobacter*, rather than the typical three-subunit (AdhA/AdhB/AdhS) structure described in *Acetobacter* and *Gluconobacter* [3]. In parallel, KSO5 retained a putative Mo-ALDH-type module comprising a molybdopterin-dependent aldehyde dehydrogenase subunit, a 2Fe-2S-binding electron-transfer protein, and a cytochrome c subunit, consistent with the three-subunit architecture reported for membrane-bound ALDH complexes in AAB [3]. The genome also encoded PqqB, PqqC, PqqD, and PqqE, whereas a clearly annotated PqqA precursor peptide was not detected, suggesting a near-complete PQQ biosynthetic module with an unresolved small-peptide annotation rather than clear absence of cofactor biosynthetic capacity. The genome further encoded dual bo3- and bd-type ubiquinol oxidases (with bd duplication), a complete NADH dehydrogenase complex I (NuoA-N; notably absent in *Gluconobacter*), succinate dehydrogenase (SdhAB) [35], and an expanded bc1 complex (PetABC) with extra copies of petB/petC. In addition, the cytochrome-c maturation locus was largely conserved as CcmABCEFGH, although CcmG was not clearly identified in the current annotation [36]. Together, these features suggest that KSO5 maintains a highly configured electron-transfer network suited to fluctuating oxygen availability and persistent acid stress during vinegar fermentation.

**Figure 12:**
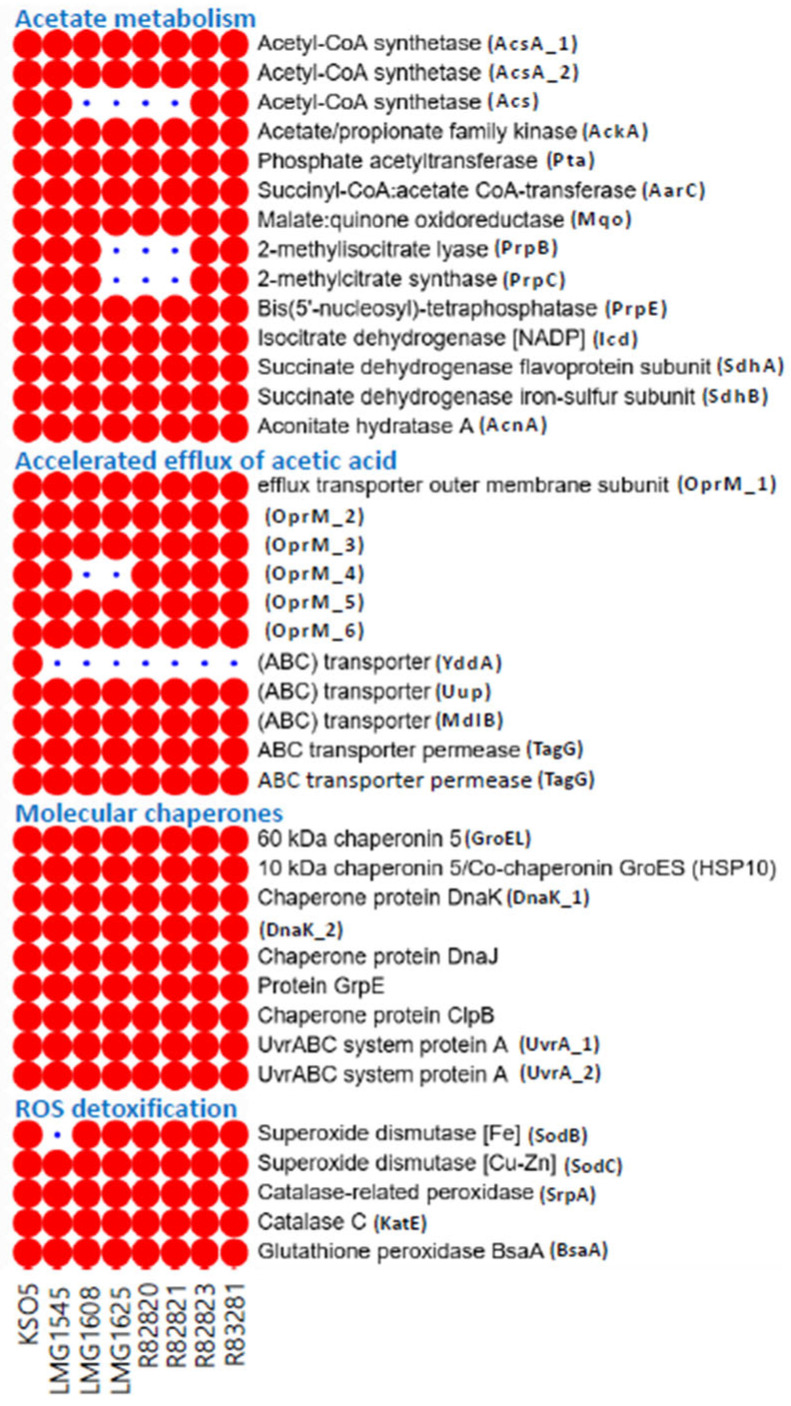
Pangenome-based presence–absence heatmap of acid-tolerance gene modules across eight *A. cerevisiae* genomes. The heatmap summarizes gene content (red = present; blue = absent) for four acid-tolerance modules across eight genomes. Columns represent strains.

In addition, *A. cerevisiae* KSO5 encoded a broad intracellular acetate activation and assimilation repertoire, including three acetyl-CoA synthetases (AcsA_1, AcsA_2, Acs), the AckA–Pta route, and the AarC–Mqo-associated modified CAC framework, together with TCA nodes (Icd, SdhAB, AcnA) and PrpB/PrpC and a PrpE-annotated locus (Figure 6C and Figure 12; Supplementary Table S1). Across the eight strains, these loci appeared predominantly red in the heatmap, with only sporadic blue gaps in non-KSO5 isolates, indicating a species-level backbone while positioning KSO5 as fully equipped at the gene-content level. This genomic pattern was consistent with prior functional studies implicating acetate activation/assimilation systems (e.g., Acs and the AckA–Pta route) and an AarC–Mqo-associated modified CAC in acetate oxidation and acetic-acid resistance in acetic acid bacteria [37,38]. In aggregate, the heatmap profile supported the interpretation that acetic-acid resilience in KSO5 reflected distributed metabolic capacity across acetate activation and central-carbon flux, rather than dependence on a single determinant [29].

The presence of respiratory-chain-linked alcohol and aldehyde oxidation modules, together with acetate-assimilation enzymes, provides a plausible genomic basis for the ability of KSO5 to maintain acid production under ethanol stress.

#### 3.8.2. Accelerated Efflux of Acetic Acid

A second pillar of acid tolerance in *A. cerevisiae* KSO5 was active acetic-acid extrusion (Figure 6D and Figure 12). Two transporter classes were commonly associated with this process: proton-motive-force-dependent efflux systems (e.g., RND/SMR/MFS families operating with TolC/OprM-like outer-membrane channels) and ATP-binding cassette (ABC) transporters [39,40,41]. To summarize the gene-content context of this efflux layer, we examined transporter loci that were (i) annotated as OprM/TolC-like outer-membrane efflux channels or ABC exporter components and (ii) resolved as distinct orthologous clusters in the pan-genome presence–absence matrix. This filtering retained six OprM-family paralogs (oprM_1–oprM_6) and five ABC transporter components (YddA, Uup, MdlB, TagG, and TagG permease) (Figure 12). In previously characterized bacterial efflux architectures, OprM-family proteins are typically described as outer-membrane conduits of tripartite assemblies that operate together with proton-motive-force-energized inner-membrane transporters, whereas ABC transporters are generally defined as ATP-driven exporters [42,43,44,45]. In KSO5, all loci within this module scored “present,” a pattern that was consistent with a gene repertoire compatible with acetic-acid efflux. Across the eight genomes, these loci were largely conserved, with only sporadic absences in non-KSO5 strains, a distribution that was indicative of a species-level backbone underlying the potential for acetic-acid extrusion (Figure 12).

The identification of PMF-driven and ABC-type transport systems suggests that KSO5 may reduce intracellular acid accumulation through active transport-based mechanisms. These systems, together with broader transporter repertoires for sugars, organic acids, and ions, may help maintain intracellular homeostasis during ethanol oxidation and extracellular acidification.

#### 3.8.3. Stress Response Molecular Chaperones

The proteostasis/repair arm is universally conserved across all eight A. cerevisiae genomes: GroES–GroEL, DnaK–DnaJ–GrpE, ClpB, and the DNA-repair factor UvrA show uninterrupted presence in every strain (Figure 6E,F and Figure 12). This species-wide invariance aligns with functional evidence in AAB—GroES/EL induction under acetic acid, ethanol, and heat correlates with improved viability [46], while dnaK, dnaJ, grpE, and clpB are upregulated during acetic-acid fermentation [47,48]; overexpressing uvrA further enhances resistance [49,50]. Together, the conservation of major molecular chaperones suggests that KSO5 retains a protein-quality-control system capable of mitigating ethanol- and acid-induced protein damage. Because ethanol and acetic acid can destabilize protein folding and membrane-associated enzymes, these chaperone systems may support the maintenance of respiratory and metabolic functions under fermentation stress.

#### 3.8.4. Oxidative-Stress Detoxification (ROS Defense)

The oxidative-stress detoxification repertoire is conserved across the eight A. cerevisiae genomes, with one exception: LMG1545 lacks SodB. In contrast, KSO5 encodes a complete set of core ROS-defense enzymes, including superoxide dismutase (Sod), catalase (KatE), the catalase-related peroxidase (SrpA), and glutathione peroxidase (BsaA) (Figure 12; Supplementary Table S1). Collectively, these enzymes are well suited to mitigate the elevated reactive oxygen species generated during membrane-bound oxidative fermentation (e.g., superoxide and hydrogen peroxide), thereby limiting oxidative damage to lipids, proteins, and DNA and potentially enhancing fitness under high acetic-acid loads (Figure 6G).

#### 3.8.5. SNP Comparison of Proteins Involved in Acetic Acid Resilience Mechanisms Across Eight *A. cerevisiae* Strains

**Figure 13:**
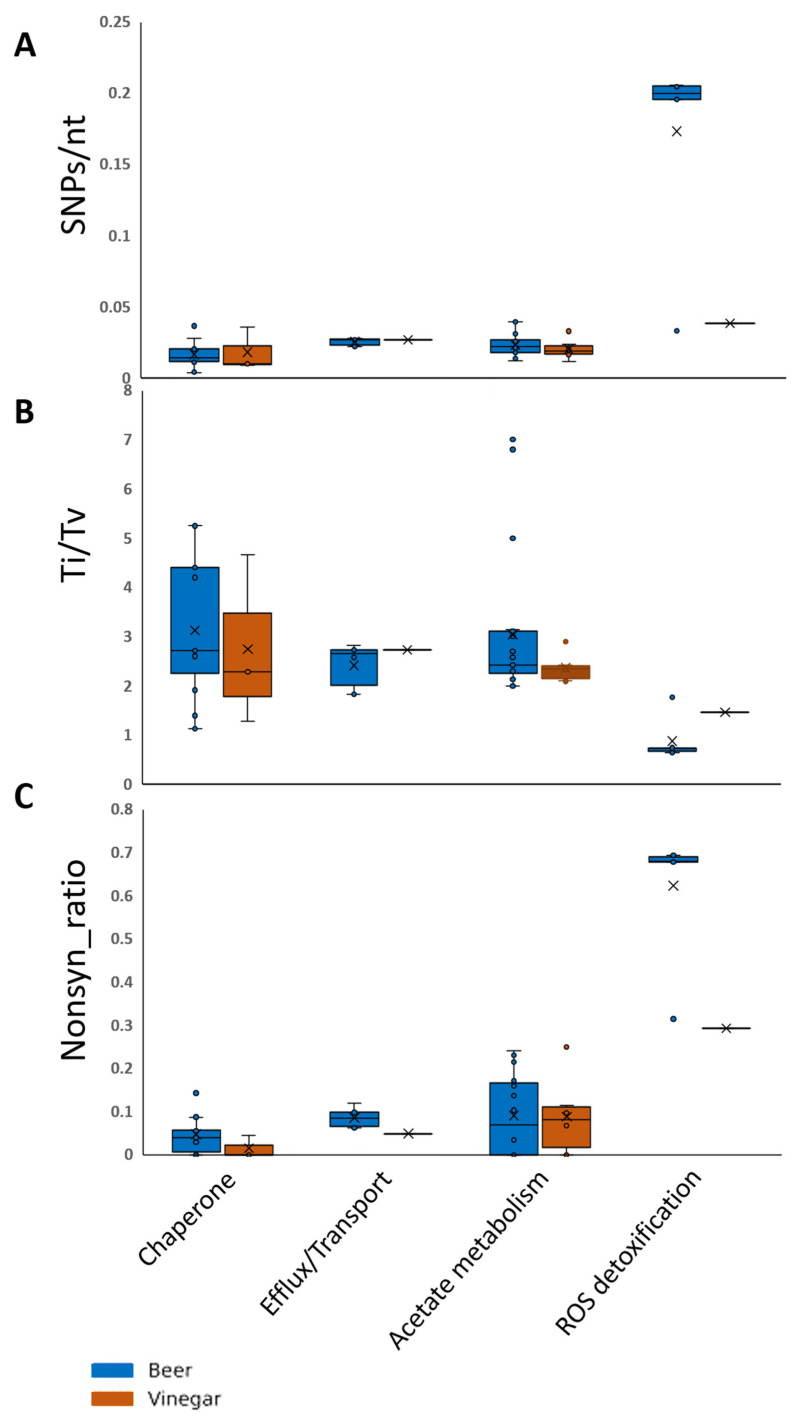
Mechanism-wise distributions of sequence variation. Boxplots summarize per-strain metrics from eight *A. cerevisiae* genomes, computed on MSA with the KSO5 allele as reference (CDSs were trimmed to the common in-frame length when needed), examining three metrics by mechanism for the Beer vs. Vinegar (=LMG1545) source groups. (**A**) SNPs per nucleotide (SNPs/nt) across each CDS. (**B**) Transition/transversion ratio (Ti/Tv). (**C**) Proportion of nonsynonymous codons among variable codons (nonsynonymous ÷ [synonymous + nonsynonymous + nonsense + stop-loss]). Mechanism assignments follow the module-representative genes. Box-and-whisker plots were generated with internal elements and outliers shown, including a mean marker and the median.

Deliverables were compiled as Figure 13, which summarized mechanism-wise distributions of SNPs/nt, Ti/Tv, and the nonsynonymous ratio across Beer- and Vinegar-origin lineages (Table 2). In the chaperone/stress-response category, median SNPs/nt remained low in both lineages (Beer 0.0141 vs. Vinegar 0.0098); Ti/Tv was higher in Beer (3.46 vs. 2.29), and the nonsynonymous fraction was near zero (0.040 vs. 0.000). In efflux/transport, SNPs/nt was similarly low in both groups (0.0267 in each), Ti/Tv was nearly indistinguishable (2.66 vs. 2.73), and the nonsynonymous fraction was modestly higher in Beer (0.0845 vs. 0.0488). In enzymatic acetate metabolism (assimilation), SNPs/nt values were close between lineages (Beer 0.0219 vs. Vinegar 0.0189), Ti/Tv remained transition-biased and similar (2.43 vs. 2.35), and nonsynonymous fractions were low in both groups (Beer 0.0685 vs. Vinegar 0.0817). By contrast, the ROS detoxification category—represented by SrpA in the sentinel panel—showed higher between-lineage separation across metrics: Beer exhibited higher SNP density (0.200 vs. 0.0384), lower Ti/Tv (0.740 vs. 1.471), and a higher nonsynonymous fraction (0.681 vs. 0.293). Overall, across this sentinel set, chaperone, efflux/transport, and central-carbon modules displayed comparatively low variation between lineages, whereas the strongest differences were concentrated in the ROS detoxification-represented category (Figure 12), consistent with heterogeneous variability across mechanisms rather than a uniform shift across the entire acid-resilience repertoire.

The SNP comparison of module-representative proteins revealed sequence-level variation among the eight *A. cerevisiae* strains in proteins associated with acetate metabolism, efflux, chaperone response, and oxidative-stress detoxification. These variations may reflect strain-level diversification of stress-resilience modules; however, their direct functional consequences cannot be inferred from sequence comparison alone. Therefore, these SNP patterns should be interpreted as candidate genomic signatures for future functional validation rather than as experimentally confirmed determinants of acetic-acid resilience.

## 4. Conclusions

In this study, we obtained the first complete circular genome sequence of *Acetobacter cerevisiae* KSO5, comprising one chromosome and two plasmids, and performed an integrated comparative genomic and phenotypic characterization of this strain. Core-genome phylogeny confirmed the placement of KSO5 within the *A. cerevisiae* lineage, while comparative analyses identified strain-specific genomic islands, mobile genetic elements, plasmid-associated modules, and distinctive COG-based functional tendencies. The genome further encoded oxidative fermentation-related enzymes and predicted acetate-handling routes that are consistent with its fermentation-associated physiology. Phenotypic evaluation showed that KSO5 maintained growth and titratable acidity production up to 9% ethanol, with the strongest performance observed at 7–9% ethanol, whereas both traits were markedly reduced at 10% ethanol. In addition, KSO5 exhibited high ethanol consumption in 5% ethanol medium, comparable to that of *A. pasteurianus* LMG 1262. Taken together, these results provide a concise genomic and phenotypic framework for understanding the fermentation-relevant characteristics of KSO5.

## Figures and Tables

**Table 3:** Plasmid-associated protein modules and genetic implications in *A. cerevisiae* strains.

Strain	Key Plasmid-Associated Protein Modules	Genetic Implication
KSO5	RepA (CyRepA1 family), CRISPR-associated primase-polymerase	Autonomous plasmid replication; potential integration of CRISPR-mediated defense and recombination modules (This study)
LMG 1625	Two RepA variants, CcdB toxin, HigB toxin, stabilization proteins	Toxin–antitoxin (TA) system-based plasmid stabilization (This study)
LMG 1545	RepB, MobA, RepC, MobC	Complete modules for plasmid partitioning, mobilization, and replication (This study)
LMG 1608	RepB, MobA	Focused on distribution and transfer rather than autonomous replication (This study)
R-82820/R-82821	RepC, MobC, stabilization proteins	Minimal replication and mobilization module architecture (This study)
R-82823/R-83281	RepC, RepB, MobA	Canonical RepABC system combining replication initiation, partitioning, and transfer (This study)

**Table 4:** General genomic features of the chromosome and plasmids of the strain KSO5.

Assortment	Name	NCBI Accession No.	Length	GC (%)	Depth	Circular	CDS	tRNA	rRNA
Chromosome	KSO5_Chr	CP172014	3,257,599	57.8	294.0	YES	2889	54	12
Plasmids	KSO5_P1	CP172015	4905	56.2	14.3	YES	5	0	0
	KSO5_P2	CP172016	4820	56.1	28.2	YES	4	0	0
	Total		3,267,324	57.8	293.2				

## Data Availability

The original contributions presented in this study are included in the article/Supplementary Material. Further inquiries can be directed to the corresponding author.

## Supplementary Materials

The following supporting information can be downloaded at: https://www.mdpi.com/article/10.3390/microorganisms14051128/s1, Supplementary Methods: Phenotyping under ethanol stress. Supplementary Figures S1–S3: Figure S1, 16S rRNA Phylogenetic tree (*n* = 20); Figure S2, 16S rRNA gene-based phylogenetic trees of acetic acid bacteria (*n* = 39); Figure S3, GTDB-based phylogenomic tree of acetic acid bacteria (*n* = 39). Supplementary Data S1 (.xlsx): Eight strains AC_Normalization_Quality_Factors. Supplementary Data S2 (.xlsx): Eight strains MGE_Analysis. Supplementary Data S3 (.xlsx): KSO5_ANI_percentage. Supplementary Data S4 (.xlsx): DDH_KSO5. Supplementary Tables S1–S3 (.docx): Table S1, Mechanisms involved in acetic acid tolerance in *A. cerevisiae* KSO5; Table S2, Membrane-bound dehydrogenases- and respiratory chain-related proteins encoded in the genome of KSO5; Table S3, Proteins involved in carbohydrates oxidation in the genome of KSO5.

## References

[1] Mamlouk D., Gullo M. (2013). Acetic acid bacteria: Physiology and carbon sources oxidation. Indian J. Microbiol..

[2] Chinnawirotpisan P., Theeragool G., Limtong S., Toyama H., Adachi O.O., Matsushita K. (2003). Quinoprotein alcohol dehydrogenase is involved in catabolic acetate production, while NAD-dependent alcohol dehydrogenase in ethanol assimilation in *Acetobacter pasteurianus* SKU1108. J. Biosci. Bioeng..

[3] Miah R., Nina S., Murate T., Kataoka N., Matsutani M., Ano Y., Matsushita K., Yakushi T. (2022). Dissection and Reconstitution Provide Insights into Electron Transport in the Membrane-Bound Aldehyde Dehydrogenase Complex of *Gluconacetobacter diazotrophicus*. J. Bacteriol..

[4] Illeghems K., De Vuyst L., Weckx S. (2013). Complete genome sequence and comparative analysis of *Acetobacter pasteurianus* 386B, a strain well-adapted to the cocoa bean fermentation ecosystem. BMC Genom..

[5] Kim S.H., Jeong W.S., Kim S.Y., Yeo S.H. (2023). Quality and Functional Characterization of Acetic Acid Bacteria Isolated from Farm-Produced Fruit Vinegars. Fermentation.

[6] Prust C., Hoffmeister M., Liesegang H., Wiezer A., Fricke W.F., Ehrenreich A., Gottschalk G., Deppenmeier U. (2005). Complete Genome Sequence of the Acetic Acid Bacterium *Gluconobacter oxydans*. Nat. Biotechnol..

[7] Tamura K., Nei M., Kumar S. (2004). Prospects for inferring very large phylogenies by using the neighbor-joining method. Proc. Natl. Acad. Sci. USA.

[8] Walker B.J., Abeel T., Shea T., Priest M., Abouelliel A., Sakthikumar S., Cuomo C.A., Zeng Q., Wortman J., Young S.K. (2014). Pilon: An Integrated Tool for Comprehensive Microbial Variant Detection and Genome Assembly Improvement. PLoS ONE.

[9] Seemann T. (2014). Prokka: Rapid prokaryotic genome annotation. Bioinformatics.

[10] Page A.J., Cummins C.A., Hunt M., Wong V.K., Reuter S., Holden M.T.G., Fookes M., Falush D., Keane J.A., Parkhill J. (2015). Roary: Rapid Large-Scale Prokaryote Pan Genome Analysis. Bioinformatics.

[11] Price M.N., Dehal P.S., Arkin A.P. (2010). FastTree 2–Approximately maximum-likelihood trees for large alignments. PLoS ONE.

[12] Letunic I., Bork P. (2021). Interactive Tree of Life (iTOL) v5: An online tool for phylogenetic tree display and annotation. Nucleic Acids Res..

[13] Grigoriev A. (1998). Analyzing genomes with cumulative skew diagrams. Nucleic Acids Res..

[14] Lobry J.R. (1996). Asymmetric substitution patterns in the two DNA strands of bacteria. Mol. Biol. Evol..

[15] Gao F., Zhang C.T. (2008). Ori-Finder: A web-based system for finding oriCs in unannotated bacterial genomes. BMC Bioinform..

[16] Kono N., Arakawa K., Tomita M. (2011). Comprehensive prediction of chromosome dimer resolution sites in bacterial genomes. BMC Genom..

[17] Mackiewicz P., Zakrzewska-Czerwińska J., Zawilak A., Dudek M.R., Cebrat S. (2004). Where does bacterial replication start? Rules for predicting the oriC region. Nucleic Acids Res..

[18] Adler P., Frey L.J., Berger A., Bolten C.J., Hansen C.E., Wittmann C. (2014). The Key to Acetate: Metabolic Fluxes of Acetic Acid Bacteria under Cocoa Pulp Fermentation-Simulating Conditions. Appl. Environ. Microbiol..

[19] García-García I., Cañete-Rodríguez A.M., Santos-Dueñas I.M., Jiménez-Hornero J.E., Ehrenreich A., Liebl W., García-Martínez T., Mauricio J.C. (2017). Biotechnologically Relevant Features of Gluconic Acid Production by Acetic Acid Bacteria. Acetic Acid Bact..

[20] Yin H., Zhang R., Xia M., Bai X., Mou J., Zheng Y., Wang M. (2017). Effect of Aspartic Acid and Glutamate on Metabolism and Acid Stress Resistance of *Acetobacter pasteurianus*. Microb. Cell Fact..

[21] Cummins J.T., King T.E., Cheldelin V.H. (1957). The biological oxidation of sorbitol. J. Biol. Chem..

[22] He Y., Xie Z., Zhang H., Liebl W., Toyama H., Chen F. (2022). Oxidative Fermentation of Acetic Acid Bacteria and Its Products. Front. Microbiol..

[23] Shinagawa E., Matsushita K., Adachi O., Ameyama M. (1982). Purification and characterization of D-sorbitol dehydrogenase from membrane of *Gluconobacter suboxydans* var. α. Agric. Biol. Chem..

[24] Argüelles J.C. (2000). Physiological roles of trehalose in bacteria and yeasts: A comparative analysis. Arch. Microbiol..

[25] Booth I.R., Edwards M.D., Black S., Schumann U., Miller S. (2007). Mechanosensitive channels in bacteria: Signs of closure?. Nat. Rev. Microbiol..

[26] Eberlein C., Baumgarten T., Starke S., Heipieper H.J. (2018). Immediate response mechanisms of gram-negative solvent-tolerant bacteria to cope with environmental stress: Cis-trans isomerization of unsaturated fatty acids and outer membrane vesicle secretion. Appl. Microbiol. Biotechnol..

[27] Mitina I., Grajdieru C., Sturza R., Mitin V., Rubtov S., Balanuta A., Behta E., Deaghileva A., Inci F., Hacıosmanoğlu N. (2025). Molecular Detection of *Acetobacter aceti* and *Acetobacter pasteurianus* at Different Stages of Wine Production. Foods.

[28] Longin C., Guilloux-Benatier M., Alexandre H. (2016). Design and Performance Testing of a DNA Extraction Assay for Sensitive and Reliable Quantification of Acetic Acid Bacteria Directly in Red Wine Using Real Time PCR. Front. Microbiol..

[29] Hua S., Wang Y., Wang L., Zhou Q., Li Z., Liu P., Wang K., Zhu Y., Han D., Yu Y. (2024). Regulatory Mechanisms of Acetic Acid, Ethanol, and High Temperature Tolerances of Acetic Acid Bacteria during Vinegar Production. Microb. Cell Fact..

[30] Arai H., Kameya M., Ishii M. (2020). Complete Genome Sequence of the Acetic Acid Bacterium *Acetobacter aceti* NBRC 14818. Microbiol. Resour. Announc..

[31] Song J., Wang J., Wang X., Zhao H., Hu T., Feng Z., Lei Z., Li W., Zheng Y., Wang M. (2022). Improving the Acetic Acid Fermentation of *Acetobacter pasteurianus* by Enhancing the Energy Metabolism. Front. Bioeng. Biotechnol..

[32] Kersters K., Lisdiyanti P., Komagata K., Swings J., Dworkin M., Falkow S., Rosenberg E., Schleifer K.H., Stackebrandt E. (2006). The family *Acetobacteraceae*. The Genera Acetobacter, Acidomonas, Asaia, Gluconacetobacter, Gluconobacter, and Kozakia.

[33] Yang H., Chen T., Wang M., Zhou J., Liebl W., Barja F., Chen F. (2022). Molecular Biology: Fantastic Toolkits to Improve Knowledge and Application of Acetic Acid Bacteria. Biotechnol. Adv..

[34] Azuma Y., Hosoyama A., Matsutani M., Furuya N., Horikawa H., Harada T., Hirakawa H., Kuhara S., Matsushita K., Fujita N. (2009). Whole-Genome Analyses Reveal Genetic Instability of *Acetobacter pasteurianus*. Nucleic Acids Res..

[35] Greenfield S., Claus G.W. (1972). Nonfunctional tricarboxylic acid cycle and the mechanism of glutamate biosynthesis in *Acetobacter suboxydans*. J. Bacteriol..

[36] Sanders C., Turkarslan S., Lee D.W., Onder O., Kranz R.G., Daldal F. (2008). The Cytochrome c Maturation Components CcmF, CcmH, and CcmI Form a Membrane-Integral Multisubunit Heme Ligation Complex. J. Biol. Chem..

[37] Yang H., Yu Y., Fu C., Chen F. (2019). Bacterial acid resistance toward organic weak acid revealed by RNA-seq transcriptomic analysis in *Acetobacter pasteurianus*. Front Microbiol..

[38] Mullins E.A., Francois J.A., Kappock T.J. (2008). A specialized citric acid cycle requiring succinyl-coenzyme A (CoA): Acetate CoA-transferase (AarC) confers acetic acid resistance on the acidophile *Acetobacter aceti*. J. Bacteriol..

[39] Nakano S., Fukaya M. (2008). Analysis of proteins responsive to acetic acid in *Acetobacter*: Molecular mechanisms conferring acetic acid resistance in acetic acid bacteria. Int. J. Food Microbiol..

[40] Qiu X., Zhang Y., Hong H. (2021). Classification of acetic acid bacteria and their acid resistant mechanism. AMB Expr..

[41] Kim S.H., Jang H.W., Park J.J., Nam D.G., Lee S.J., Yeo S.H., Kim S.Y. (2024). Antibiotic resistance in acetic acid bacteria originating from vinegar. Antibiotics.

[42] Li X.Z., Nikaido H. (2009). Efflux-Mediated Drug Resistance in Bacteria: An Update. Drugs.

[43] Neuberger A., Du D., Luisi B.F. (2018). Structure and mechanism of bacterial tripartite efflux pumps. Res. Microbiol..

[44] Higgins C.F. (2007). Multiple molecular mechanisms for multidrug resistance transporters. Nature.

[45] Davidson A.L., Dassa E., Orelle C., Chen J. (2008). Structure, function, and evolution of bacterial ATP-binding cassette systems. Microbiol. Mol. Biol. Rev..

[46] Wang B., Shao Y., Chen T., Chen W., Chen F. (2015). Global insights into acetic acid resistance mechanisms and genetic stability of *Acetobacter pasteurianus* strains by comparative genomics. Sci. Rep..

[47] Andrés-Barrao C., Saad M.M., Chappuis M.L., Boffa M., Perret X., Ortega Pérez R., Barja F. (2012). Proteome Analysis of *Acetobacter pasteurianus* during Acetic Acid Fermentation. J. Proteom..

[48] Okamoto-Kainuma A., Ishikawa M., Nakamura H., Fukazawa S., Tanaka N., Yamagami K., Koizumi Y. (2011). Characterization of *rpoH* in *Acetobacter pasteurianus* NBRC3283. J. Biosci. Bioeng..

[49] Zheng Y., Wang J., Bai X., Chang Y., Mou J., Song J., Wang M. (2018). Improving the Acetic Acid Tolerance and Fermentation of *Acetobacter pasteurianus* by Nucleotide Excision Repair Protein UvrA. Appl. Microbiol. Biotechnol..

[50] Kim S.H., Kim J.Y., Jeong W.S., Gwon H.M., Kim S.Y., Yeo S.H. (2022). Culture and Function-Related Characteristics of Six Acetic Acid Bacterial Strains Isolated from Farm-Made Fermented Vinegars. Korean J. Food Preserv..

