# Supplementary material for: Complete Genome Sequence and Comparative Genomics of Acetobacter cerevisiae KSO5 (KACC 92352P) Provide Genome-Based Insights into Acid Tolerance
Source: Microorganisms. 2026 May 15;14(5):1128. doi: 10.3390/microorganisms14051128 (PMC13209655; doi:10.3390/microorganisms14051128)
Supplement: Supplementary file 1 [file microorganisms-14-01128-s001.zip › KSO5_Supplementary Figure(1-3).pdf]

SUPPLEMENTARY FIGURE MATERIALS

Complete genome sequence and comparative genomics of *Acetobacter cerevisiae* KSO5 (KACC 92352P) provide genome-based insights into acid tolerance

Sun Hee Kim[1], Dae Gyu Choi[2], Dong Min Han[2], Seong-Eui Yoo[1], Jin Ju Park[1], Chan Woo Kim[1], and So-Young Kim[1]*

[1]Fermented and Processed Food Research Division, Department of Food Sciences, NICS, RDA, Wanju, 55365, Korea; sunheekim00@korea.kr (S.H.K.); dbtjddml@korea.kr (S.-E.Y); waemma25@korea.kr (J.J.P.); kcw5142@korea.kr (C.W.K.)

[2]Department of Life Science, Chung-Ang University, Seoul 06974, Republic of Korea; chleorb21@cau.ac.kr (D.G.C); gonigori@naver.com (D.M.H)

[*]Correspondence: foodksy@korea.kr (S.-Y.K.); Tel.: +82-63-238-3610; Fax.: +82-63-238-3843

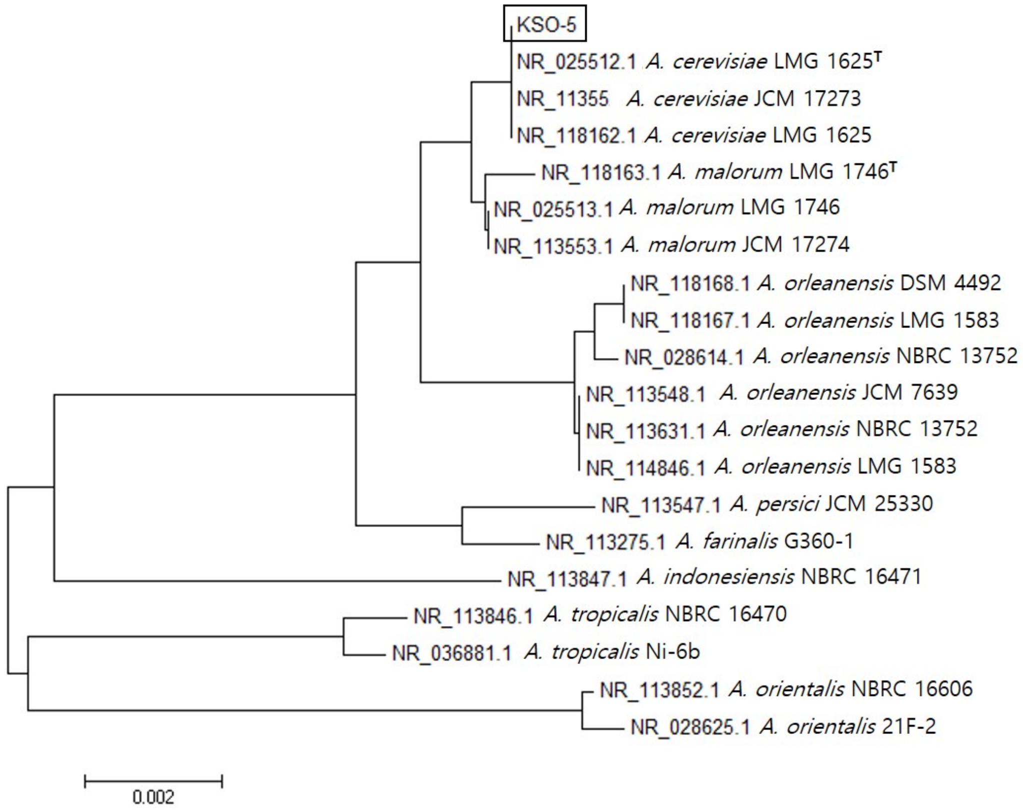

Supplementary Figure S1.

Neighbor-joining phylogeny of 16S rRNA sequences (n=20) placing strain KSO5 within Acetobacter; distances (substitutions/site) were estimated by the Maximum Composite Likelihood method in MEGA6 [7].

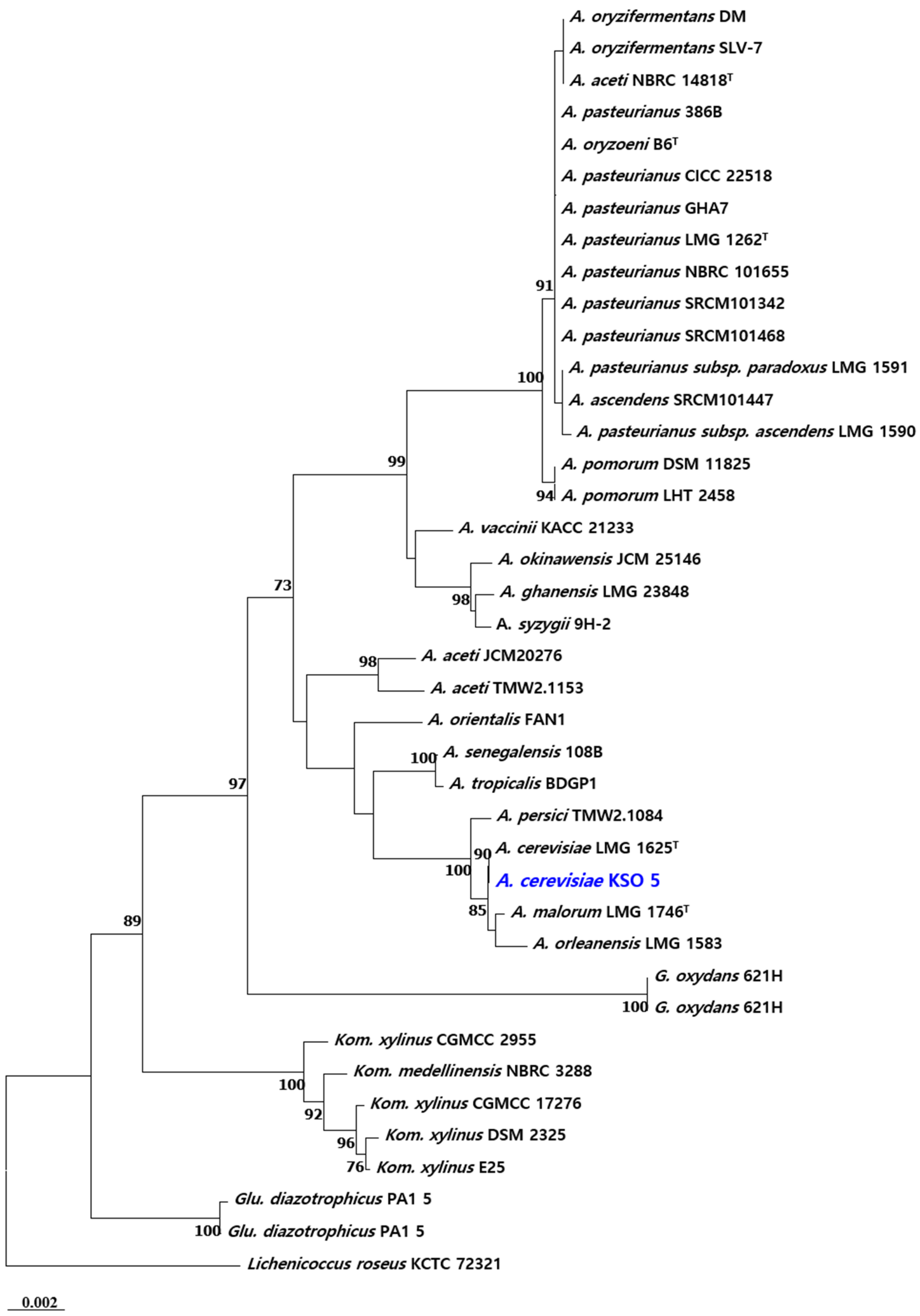

Supplementary Figure S2.

16S rRNA gene-based phylogenetic trees of acetic acid bacteria.

Phylogenetic trees based on 16S rRNA gene sequences were reconstructed using the neighbor-joining, maximum-likelihood, and maximum-parsimony methods in MEGA 7. The sequences were aligned using Infernal v1.14. Branch support values were calculated from 1,000 bootstrap replicates. *Lichenicoccus roseus* KCTC 72321 was used as the outgroup. Scale bars indicate nucleotide substitutions per site, except for the maximum-parsimony tree, where the scale indicates the number of character-state changes.

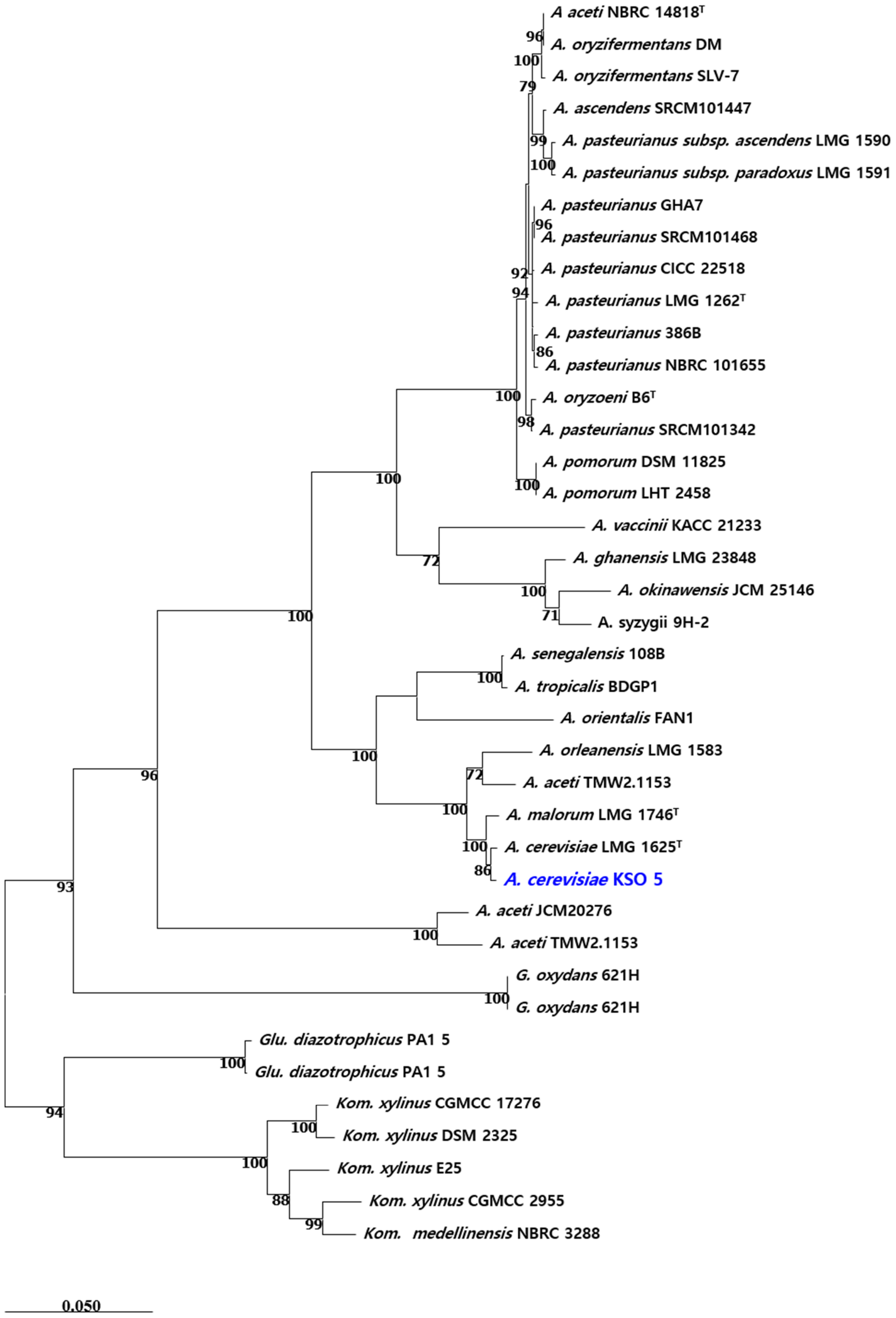
A aceti NBRC 14818T
A. oryzifermentans DM
A. oryzifermentans SLV-7
A. ascendens SRCM101447
A. pasteurianus subsp. ascendens LMG 1590
A. pasteurianus subsp. paradoxus LMG 1591
A. pasteurianus GHA7
A. pasteurianus SRCM101468
A. pasteurianus CICC 22518
A. pasteurianus LMG 1262T
A. pasteurianus 386B
A. pasteurianus NBRC 101655
A. oryzoeni B6T
A. pasteurianus SRCM101342
A. pomorum DSM 11825
A. pomorum LHT 2458
A. vaccinii KACC 21233
A. ghanensis LMG 23848
A. okinawensis JCM 25146
A. syzygii 9H-2
A. senegalensis 108B
A. tropicalis BDGP1
A. orientalis FAN1
A. orleanensis LMG 1583
A. aceti TMW2.1153
A. malorum LMG 1746T
A. cerevisiae LMG 1625T
A. cerevisiae KSO 5
A. aceti JCM20276
A. aceti TMW2.1153
G. oxydans 621H
G. oxydans 621H
Glu. diazotrophicus PA1 5
Glu. diazotrophicus PA1 5
Kom. xylinus CGMCC 17276
Kom. xylinus DSM 2325
Kom. xylinus E25
Kom. xylinus CGMCC 2955
Kom. medellinensis NBRC 3288
0.050

Supplementary Figure S3.

GTDB-based phylogenomic tree of acetic acid bacteria.

Maximum-likelihood phylogenomic tree reconstructed from the alignment of 120 conserved housekeeping proteins obtained using GTDB-Tk. The tree was constructed using MEGA 7. Bootstrap values are shown at the nodes. The scale bar indicates substitutions per site. *Lichenicoccus roseus* KCTC 72321 was used as the outgroup for rooting but is not shown in the final tree for clarity.
