# Supplementary material for: Complete Genome Sequence and Comparative Genomics of Acetobacter cerevisiae KSO5 (KACC 92352P) Provide Genome-Based Insights into Acid Tolerance
Source: Microorganisms. 2026 May 15;14(5):1128. doi: 10.3390/microorganisms14051128 (PMC13209655; doi:10.3390/microorganisms14051128)
Supplement: Supplementary file 1 [file microorganisms-14-01128-s001.zip › KSO5_Supplementary Methods.pdf]

SUPPLEMENTARY Methods MATERIALS

Complete genome sequence and comparative genomics of *Acetobacter cerevisiae* KSO5 (KACC 92352P) provide genome-based insights into acid tolerance

Sun Hee Kim[1], Dae Gyu Choi[2], Dong Min Han[2], Seong-Eui Yoo[1], Jin Ju Park[1], Chan Woo Kim[1], and So-Young Kim[1]*

[1]Fermented and Processed Food Research Division, Department of Food Sciences, NICS, RDA, Wanju, 55365, Korea; sunheekim00@korea.kr (S.H.K.); dbtjddml@korea.kr (S.-E.Y); waemma25@korea.kr (J.J.P.); kcw5142@korea.kr (C.W.K.)

[2]Department of Life Science, Chung-Ang University, Seoul 06974, Republic of Korea; chleorb21@cau.ac.kr (D.G.C); gonigori@naver.com (D.M.H)

*Correspondence: foodksy@korea.kr (S.-Y.K.); Tel.: +82-63-238-3610; Fax.: +82-63-238-3843

Supplementary Methods (M1)

The phenotyping procedures were based on previously described methods [5, 50], with minor modifications as described below.

M1.1. Strains used for comparative phenotyping

The focal strain in this study was *Acetobacter cerevisiae* KSO5 (KACC 92352P). Comparative phenotyping included the *A. cerevisiae* type strain LMG 1625 as the primary conspecific reference and representative vinegar-associated *Acetobacter* strains, including the *A. pasteurianus* type strain LMG 1262, *A. aceti* NBRC 14818, and the *A. malorum* type strain LMG 1746. The reference strains were purchased from the Korean Agricultural Culture Collection (KACC) and the Korean Collection for Type Cultures (KCTC).

M1.2. Ethanol-stress culture conditions and inoculum standardization

Acetic acid bacteria (AAB) were cultured in a liquid medium containing yeast extract (5 g/L), glucose (5 g/L), glycerol (1%, v/v), $MgSO_4 \cdot 7H_2O$ (0.2 g/L), and acetic acid (1%, v/v). Ethanol was added to final concentrations of 5%, 7%, 9%, or 10% (v/v). All strains were standardized

to an inoculum density of OD_{660}=0.5, and cultures were inoculated at 1:1000 (v/v). Fermentations were conducted in 250 mL Erlenmeyer flasks containing 50 mL medium and incubated aerobically at 30 °C with shaking at 150 rpm for up to 10 days.

M1.3. Sampling schedule

For ethanol-stress time-course profiling, samples were collected on days 1, 4, 6, 8, and 10 for measurements of growth (OD_{660}) and titratable acidity. For ethanol consumption profiling, residual ethanol concentrations were measured at the same time points in medium supplemented with 5% ethanol.

M1.4. Growth measurement (OD_{660})

Bacterial growth was monitored by measuring optical density at 660 nm (OD_{660}) using a spectrophotometer (Gen5TM, BioTek, Winooski, VT, USA). Samples were mixed thoroughly prior to measurement to minimize heterogeneity. OD_{660} values were recorded at each designated time point.

M1.5. Determination of titratable acidity

Titratable acidity was determined by neutralization titration with 0.1 N NaOH using 1% (w/v) phenolphthalein as an indicator. The endpoint was defined as the appearance of a persistent pale pink color. Titratable acidity (%) was calculated as:

Titratable acidity (%) = [0.1 N NaOH (mL) × 0.006 × 100] / sample amount (mL)

where 0.006 is the acetic acid equivalent factor. Measurements were performed at the designated time points (days 1, 4, 6, 8, and 10).

M1.6. Determination of strain-specific ethanol consumption capacity

Ethanol consumption was determined by measuring the residual ethanol concentration in medium supplemented with 5% ethanol. At each sampling time point, ethanol was distilled from the culture broth using a steam distillation system for alcohol determination (VAPODEST,

C. Gerhardt GmbH & Co. KG, Königswinter, Germany), and the ethanol concentration was measured using an alcohol densitometer (DA-155, Kyoto Electronics Manufacturing Co., Ltd., Kyoto, Japan). The values were corrected against time-matched uninoculated controls. Ethanol consumption rate was calculated as follows: ethanol consumption rate (%) = [(residual ethanol concentration in the control − residual ethanol concentration in the inoculated culture) / residual ethanol concentration in the control] × 100.

M1.7. Statistical analysis

Statistical analyses were performed for all quantitative phenotypic assays, including bacterial growth, titratable acidity, and ethanol consumption capacity. Unless otherwise stated, all quantitative phenotypic assays were performed using four independent biological replicates derived from two independent experiments, with two biological cultures per experiment ($n = 4$). Data are presented as means ± standard deviation (SD) for each sampling time point. Statistical differences among strains were assessed by one-way ANOVA followed by Dunnett's multiple-comparison test, with *Acetobacter cerevisiae* KSO5 used as the reference strain. Differences were considered statistically significant at $p < 0.05$.
