# Supplementary material for: Complete Genome Sequence and Comparative Genomics of Acetobacter cerevisiae KSO5 (KACC 92352P) Provide Genome-Based Insights into Acid Tolerance
Source: Microorganisms. 2026 May 15;14(5):1128. doi: 10.3390/microorganisms14051128 (PMC13209655; doi:10.3390/microorganisms14051128)
Supplement: Supplementary file 1 [file microorganisms-14-01128-s001.zip › KSO5_Supplementary TABLE(1-3).pdf]

SUPPLEMENTARY TABLE MATERIALS

Complete genome sequence and comparative genomics of *Acetobacter cerevisiae* KSO5 (KACC 92352P) provide genome-based insights into acid tolerance

Sun Hee Kim[1], Dae Gyu Choi[2], Dong Min Han[2], Seong-Eui Yoo[1], Jin Ju Park[1], Chan Woo Kim[1], and So-Young Kim[1]*

[1]Fermented and Processed Food Research Division, Department of Food Sciences, NICS, RDA, Wanju, 55365, Korea; sunheekim00@korea.kr (S.H.K.); dbtjddml@korea.kr (S.-E.Y); waemma25@korea.kr (J.J.P.); kcw5142@korea.kr (C.W.K.)

[2]Department of Life Science, Chung-Ang University, Seoul 06974, Republic of Korea; chleorb21@cau.ac.kr (D.G.C); gonigori@naver.com (D.M.H)

[*]Correspondence: foodksy@korea.kr (S.-Y.K.); Tel.: +82-63-238-3610; Fax.: +82-63-238-3843

Supplementary Table S1

Mechanisms involved in acetic acid tolerance in *A. cerevisiae* KSO5.

Characteristics	Proteins	Proteins ID
**Enzymatic acetate metabolism (assimilation)**		
Acetyl-CoA synthetase	AcsA_1	WP_062274159.1
Acetyl-CoA synthetase	AcsA_2	WP_395494860.1
Acetyl-CoA synthetase	Acs	WP_395496437.1
Acetate/propionate family kinase	AckA	WP_395494723.1
Phosphate acetyltransferase	Pta	WP_395494724.1
Succinyl-CoA:acetate CoA-transferase	AarC	WP_062140057.1
Malate:quinone oxidoreductase	Mqo	WP_062140455.1
2-methylisocitrate lyase	PrpB	WP_395495234.1
2-methylcitrate synthase	PrpC	WP_395495235.1
Bis(5'-nucleosyl)-tetraphosphatase	PrpE	WP_395496216.1
Isocitrate dehydrogenase [NADP]	Icd	WP_395495968.1
Succinate dehydrogenase flavoprotein subunit	SdhA	WP_062141170.1
Succinate dehydrogenase iron-sulfur subunit	SdhB	WP_062141168.1
Aconitate hydratase A	AcnA	WP_062140981.1
**Accelerated efflux of acetic acid**		
efflux transporter outer membrane subunit	OprM_1	WP_395495518.1
	OprM_2	WP_395496388.1
	OprM_3	WP_062141820.1
	OprM_4	WP_395494574.1
	OprM_5	WP_395496409.1
	OprM_6	MCP1245046.1
(ABC) transporter		WP_395494905.1
(ABC) transporter		WP_395496332.1
(ABC) transporter		WP_395496168.1
ABC transporter permease		WP_395495256.1
		WP_252353899.1
**Molecular chaperones**		
60 kDa chaperonin 5	GroEL	WP_062143938.1
10 kDa chaperonin 5/Co-chaperonin GroES (HSP10)	GroES	WP_043550518.1
Chaperone protein DnaK	DnaK_1	WP_395495535.1
	DnaK_2	WP_061492219.1
Chaperone protein DnaJ	DnaJ	WP_062248507.1
Protein GrpE	GrpE	WP_395495330.1
Chaperone protein ClpB	ClpB	WP_395494999.1
UvrABC system protein A	UvrA_1	WP_395495485.1
UvrABC system protein A	UvrA_2	WP_395494648.1
**ROS detoxification**		
Superoxide dismutase [Fe]	SodB	WP_062141943.1
Superoxide dismutase [Cu-Zn]	SodC	WP_062144375.1
Catalase-related peroxidase	SrpA	WP_395495951.1
Catalase C	KatE	WP_395495889.1
Glutathione peroxidase BsaA	BsaA	WP_215753848.1

Supplementary Table S2

Membrane-bound dehydrogenases- and respiratory chain-related proteins encoded in the genome of AC KSO5.

Characteristics	Proteins	Protein ID
**PQQ-ADH and Mo-ALDH**		
Alcohol dehydrogenase	AdhA	WP_062141631.1
	AdhB_1	WP_062141657.1
Molybdopterin dependent aldehyde dehydrogenase (Mo-ALDH)	AldhF	WP_395496179.1
(2Fe-2S)-binding protein	IorA_1	WP_062142092.1
cytochrome c	NicB	WP_062142093.1
PQQ synthesis protein	PqqB	WP_395495169.1
	PqqC	WP_395495170.1
	PqqD	WP_062141737.1
	PqqE	WP_395496431.1
**Membrane-bound oxidoreductases and terminal oxidases**		
Cytochrome bo(3) ubiquinol oxidase subunit 2	CyoA	WP_043549228.1
Ubiquinol oxidase subunit 1	CyoB	WP_062141628.1
Cytochrome bo(3) ubiquinol oxidase subunit 3	CyoC	WP_043549225.1
Cytochrome bo(3) ubiquinol oxidase subunit 4	CyoD	WP_062250477.1
Cytochrome bd-I ubiquinol oxidase subunit 1	CydA_1	WP_062250527.1
Cytochrome bd-I ubiquinol oxidase subunit 1	CydA_2	WP_062270981.1
Cytochrome bd-I ubiquinol oxidase subunit 2	CydB_1	WP_062250526.1
Cytochrome bd-I ubiquinol oxidase subunit 2	CydB_2	GBQ08435.1
**Respiratory chain core system**		
NADH-quinone oxidoreductase subunit A	NuoA	WP_395494938.1
NADH-quinone oxidoreductase subunit B	NuoB	WP_395494937.1
NADH-quinone oxidoreductase subunit C	NuoC	WP_395494936.1
NADH-quinone oxidoreductase subunit D	NuoD	WP_062141875.1
NADH-quinone oxidoreductase subunit E	NuoE	WP_395494935.1
NADH-quinone oxidoreductase subunit F	NuoF	WP_395494934.1
NADH-quinone oxidoreductase subunit G	NuoG	WP_395494933.1
NADH-quinone oxidoreductase subunit H	NuoH	WP_062141871.1
NADH-quinone oxidoreductase subunit I	NuoI	WP_043550664.1
NADH-quinone oxidoreductase subunit J	NouJ	WP_082779536.1
NADH-quinone oxidoreductase subunit K	NuoK	WP_043550666.1
NADH-quinone oxidoreductase subunit L	NuoL	WP_395494932.1
NAD(P)H-quinone oxidoreductase chain 4	NuoM	WP_395496043.1
NADH-quinone oxidoreductase subunit N	NuoN	WP_395494931.1
Succinate dehydrogenase flavoprotein subunit	SdhA	WP_062141170.1
Succinate dehydrogenase flavoprotein subunit	SdhB	WP_062141168.1
Ubiquinol:cytochrome c oxidoreductase/ Ubiquinol-cytochrome c reductase iron-sulfur subunit	PetA	WP_395495406.1
Cytochrome b6	PetB_1	WP_395496385.1
Cytochrome b	PetB_2	WP_395495404.1
Cytochrome c1	PetC_1	WP_395496079.1
Cytochrome c1	PetC_2	WP_395495405.1
Cytochrome c6	CccA	WP_395496445.1
Cytochrome c	CccA	WP_395495923.1,
		WP_395495383.1
		WP_253556341.1
		WP_062143320.1
	Cyc7	WP_231866175.1
	CycA	WP_062249023.1
	CcO	WP_395494965.1
Cytochrome c biogenesis ATP-binding export protein CcmA	CcmA	MCP1245816.1
Heme exporter protein B	CcmB	WP_082779376.1
Heme exporter protein C	CcmC	WP_061492022.1
Cytochrome c-type biogenesis protein CcmE	CcmE	WP_395496108.1
Cytochrome c-type biogenesis protein CcmF	CcmF	WP_395496107.1
Cytochrome c-type biogenesis protein CcmH	CcmH	WP_197463903.1

Supplementary Table S3

Proteins involved in carbohydrates oxidation in the genome of AC KSO5.

Characteristic	Proteins	Proteins ID
**Pentose phosphate pathway**		
Glucose-6-phosphate 1-dehydrogenase	Zwf_1(G6PD)	WP_395495500.1
	Zwf_2	WP_395495054.1
6-Phosphogluconate dehydrogenase	YqeC (6PGD)	WP_395495724.1
**PQQ-, FAD and NAD-dependent dehydrogenases**		
Glucose dehydrogenase		WP_062248189.1, WP_395495975.1, WP_062249529.1
D-sorbitol dehydrogenase		WP_062250107.1
Polyol:NADP oxidoreductase (mannitol dehydrogenase)	Por	WP_395496180.1
**Glycerol pathway**		
Glycerol kinase	GlpK	WP_062140366.1
Glycerol-3-phosphate dehydrogenase	GpsA	WP_395495015.1
D-erythritol 1-phosphate dehydrogenase	EryB (GlpD)	WP_395496326.1
Glycerol uptake facilitator protein	GlpF	WP_062140368.1
Phosphatidylserine synthase	PssA	WP_052403961.1
Phosphatidylserine decarboxylase	Psd	WP_395496066.1
**EMP pathway**		
Triosephosphate isomerase	TpiA	WP_043552678.1
Glyceraldehyde-3-phosphate dehydrogenase	GapA	WP_395495504.1
Phosphoglycerate kinase	Pgk	WP_062141162.1
2,3-bisphosphoglycerate-independent phosphoglycerate mutase	GpmI	WP_395496153.1
2,3-bisphosphoglycerate-dependent phosphoglycerate mutase	GpmA	WP_062142142.1
Enolase	Eno	WP_062272717.1
Pyruvate-flavodoxin oxidoreductase	NifJ	WP_395494728.1
**Fatty acid pathway**		
Acetyl-CoA carboxylase, biotin carboxyl carrier protein	AccB	WP_395494889.1
Acetyl-CoA carboxylase, biotin carboxyl carrier protein	AccB_1	WP_062140969.1
Acetyl-CoA carboxylase, biotin carboxyl carrier protein	AccB_2	WP_395495422.1
Acetyl-CoA carboxylase biotin carboxylase	AccC_1	WP_062140967.1
Acetyl-CoA carboxylase biotin carboxylase	AccC_2	WP_395494890.1
Acetyl-CoA carboxylase biotin carboxylase	AccC_3	WP_395495423.1
Malonyl CoA-acyl carrier protein transacylase	FabD	WP_395496290.1
3-oxoacyl-[acyl-carrier-protein] reductase FabG	FabG_1	WP_395496278.1
	FabG_2	WP_395496289.1
	FabG_3	WP_395494815.1
	FabG_4	WP_395494859.1
Enoyl-[acyl-carrier-protein] reductase [NADH] FabI	FabI	WP_061509200.1
**Trehalose synthesis and utilization**		
Trehalose-6-phosphate synthase	OtsA	WP_062144274.1
Trehalose-6-phosphate phosphatase	OtsB	WP_062273541.1
Malto-oligosyltrehalose trehalohydrolase (synthase)	TreZ	WP_395494833.1
Periplasmic trehalase	TreA	WP_395496447.1
Trehalase	Sga1	WP_395494539.1
**Mechanosensitive ion channels**		
Large-conductance mechanosensitive channel	MscL	WP_062140014.1
Small-conductance mechanosensitive channel	MscS	WP_197461360.1
		WP_197461360.1
		WP_062142434.1
		WP_395495462.1
Small-conductance mechanosensitive channel	MscK	WP_395496005.1
